# Enhanced Taylor–Aris dispersion in slender pulsatile annular channels: implications for perivascular solute transport

**DOI:** 10.1017/jfm.2026.11573

**Published:** 2026-05-26

**Authors:** Drik Sarkar, Saikat Mukherjee

**Affiliations:** 1 Department of Mechanical Engineering, Iowa State University, Ames, IA 50011, USA

**Keywords:** biological fluid dynamics, flow-vessel interactions, dispersion

## Abstract

We derive expressions for the long-time effective dispersion coefficients of a solute in a slender annular channel with a spatiotemporally pulsating inner boundary. The problem is motivated by transport in perivascular spaces (PVSs) that are subjected to pulsations induced by travelling waves in the brain. Compared with steady flow, pulsations enhance the effective diffusivity and solute drift which scale quadratically with the wave amplitude, and depend on the ratio of wave-induced to bulk-flow-induced Péclet numbers, 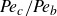 . The mean enhancement in diffusivity can be decomposed into a bulk-flow-induced contribution and two wave-induced corrections: entropic slowdown, which reduces diffusivity due to solute lodging in the constrictions, for 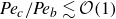 , and shuttle dispersion which enhances diffusivity due to oscillatory solute transport for 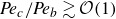 . The pulsations also induce an effective solute drift, that scales quadratically with the wave amplitude and linearly with 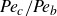 . The effective dispersion coefficients are sensitive to the annular cross-sectional area ratio with narrower geometries yielding stronger enhancements. For a representative murine PVS geometry subjected to pulsations under delta wave parameters, the mean enhancement of diffusivity, normalised by its value in steady flow, is 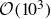 and the mean enhancement in effective solute drift is 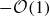 . Physiological mechanisms such as frequency-dependent wave amplitude, and approximately constant wave velocity across brain travelling waves, may diminish the enhancement magnitudes. The research presents a generalised framework for quantifying dispersion in spatiotemporally varying annular conduits and improves our understanding of perivascular solute transport.

## Graphical abstract

**Figure:**
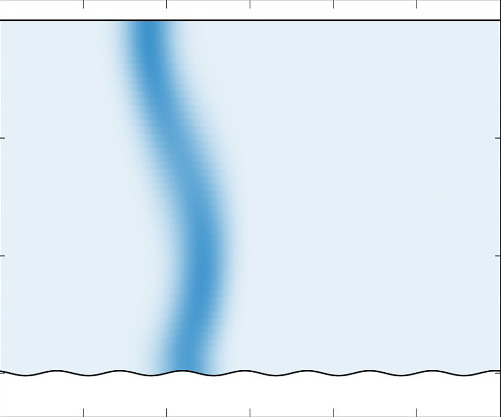

## 1. Introduction

Fluid flow and solute dispersion through slender annular conduits is fundamental to various biological and engineered systems. Examples include microfluidic devices (Zhao & Bau 2007; Hardoüin *et al.* 2020), catheter flows (Dash, Jayaraman & Mehta 1996; Sarkar & Jayaraman 2001), transport in xylem and phloem (Nakad *et al.* 2021), flow in bones (Cowin & Cardoso 2015), the subarachnoid space in the brain, spinal chord and optic nerve sheath (Loth, Yardimci & Alperin 2001; Sánchez *et al.* 2018; Salerno, Cardillo & Camporeale 2020; Rossinelli *et al.* 2024) and perivascular spaces (PVSs) in the brain (Iliff *et al.* 2012; Xie *et al.* 2013; Jessen *et al.* 2015; Rasmussen, Mestre & Nedergaard 2018; Nedergaard & Goldman 2020; Kelley 2021). A characteristic feature of the above geometries is that the axial extent considerably exceeds the radial dimension, leading to a large aspect ratio. As a result, solute transport can often be described as an effective dispersion in the axial direction, referred to as Taylor or Taylor–Aris dispersion (Taylor 1953; Aris 1956; Frankel & Brenner 1989; Mercer & Roberts 1994). The shearing effect of fluid flow leads to fast diffusive transport and rapid homogenisation across the cross-section, resulting in an enhanced effective diffusion coefficient along the axial direction. This enhanced diffusion coefficient typically scales as 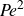 , where 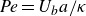 is the Péclet number quantifying the ratio of diffusion and advection timescales, expressed in terms of the mean fluid velocity 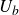 , the radial dimension 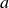 and the diffusion coefficient of the solute 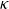 (Taylor 1953; Aris 1956). Taylor dispersion has been extensively studied across a range of configurations, including cylindrical tubes (Taylor 1954; Mercer & Roberts 1990, 1994), annular geometries (Sankarasubramanian & Gill 1971; Fallon & Chauhan 2005; Paul 2009; Chu *et al.* 2020) and more complex domains (Frankel & Brenner 1991; Rosencrans 1997), with emphasis on understanding how the flow conditions and geometry modulate solute transport.

Spatiotemporal fluctuations in channel boundaries have been found to significantly impact solute dispersion (Marbach & Alim 2019; Chakrabarti & Saintillan 2020; Chang & Santiago 2023; Chang & Santiago 2023; Wang *et al.* 2023; Alexandre, Guérin & Dean 2025). These fluctuations can be induced by wall deformation, peristalsis, arbitrary spatial variations and absorption in the channel walls, and can alter the transient concentration profiles that shape long-term dispersion behaviour. Two important mechanisms have been identified that modulate dispersion in the presence of spatiotemporal pulsations (Marbach, Dean & Bocquet 2018; Marbach & Alim 2019). The first mechanism is entropic slowdown, which acts as a negative contribution to the effective dispersion (Martens *et al.* 2013; Yang *et al.* 2017; Marbach *et al.* 2018; Marbach & Alim 2019). As the channel walls oscillate in space, solute particles get trapped in the cavities and constrictions created in the channel boundaries, making it difficult to traverse along the channel, and consequently counteracting dispersion. The second mechanism is shuttle dispersion, which arises from the alternating shear profile created due to the pulsations (Watson 1983; Schmidt, McCready & Ostafin 2005; Marbach & Alim 2019). The oscillating axial flow induced by the pulsations transport solutes back and forth and positively aids the diffusive spread, enhancing effective dispersion. The relative dominance of these two mechanisms is modulated by the wavelength and frequency of the travelling fluctuations and the bulk-flow speed in the channel (Marbach *et al.* 2018; Marbach & Alim 2019). Particularly, this modulation of solute dispersion is critical in biological flows, where vessels and surrounding spaces undergo regular pulsations. Dispersion in fluctuating annular channels has received comparatively less attention than cylindrical channels (Aris 1959; Sankarasubramanian & Gill 1971; Tsangaris & Athanassiadis 1985; Kumar Roy, Saha & Debnath 2017; Chu *et al.* 2020), although they are prevalent across various biological contexts like PVSs in the brain (Mestre *et al.* 2018; Tithof *et al.* 2019, 2022; Kelley 2021; Troyetsky *et al.* 2021; Kelley & Thomas 2023).

The PVSs are near-annular conduits that line the brain’s vasculature, forming an efficient transport pathway for cerebrospinal fluid (CSF) and metabolic waste. While PVSs are realistically eccentric, elliptical and imperfect annuli (Mestre *et al.* 2018; Tithof *et al.* 2019; Vinje, Bakker & Rognes 2021), the slow and viscous nature of the flow is amenable to simplified annular assumptions with an effective hydraulic resistance (Tithof *et al.* 2019), particularly in reduced-order models of PVSs (Daversin-Catty, Gjerde & Rognes 2022; Tithof *et al.* 2022; Mukherjee, Mirzaee & Tithof 2023). These approximations are convenient since PVSs are longer compared with their width, resulting in an aspect ratio (ratio of length to the width) that is sufficiently large (Daversin-Catty *et al.* 2020, 2022; Tithof *et al.* 2022; Mukherjee *et al.* 2023). Interestingly, PVSs undergo dynamic spatiotemporal fluctuations in their geometry due to the fluctuations in the compliant vessel wall that forms their inner boundary. These deformations arise from travelling waves in different phases of sleep, cardiac pulsations and acute ionic waves in seizures and spreading depression (Mestre *et al.* 2018; Mestre *et al.* 2020; Bojarskaite *et al.* 2023; Mukherjee *et al.* 2023). Solute dispersion behaviour within the PVS, in the presence of these pulsations, is critical for understanding the details of the CSF-mediated waste clearance mechanism in the central nervous system (also known as the ‘glymphatic system’), because impaired clearance has been linked to the development of neurodegenerative conditions such as Alzheimer’s disease (Iliff *et al.* 2012; Xie *et al.* 2013; Mukherjee & Tithof 2022; Watkins, Mukherjee & Tithof 2024).

Historically, several analytical approaches have been developed to quantify Taylor dispersion through slender channels exhibiting spatial or spatiotemporal fluctuations. While Taylor’s seminal work on shear-induced diffusion enhancement was rooted in mathematical intuitions and experiments (Taylor 1953), Aris formalised the theory based on the method of moments (Aris 1956, 1959). Subsequent developments include asymptotic expansions (Chatwin 1970), generalised Taylor dispersion theory introduced by Frankel and Brenner (Frankel & Brenner 1989, 1991; Chakrabarti & Saintillan 2020), method of moments with Dirac’s bra–ket formalism (Vedel & Bruus 2012), Lagrangian approaches incorporating Ficks–Jacobs equations (Martens *et al.* 2013), Kubo-type formulas using the generalised Fokker–Planck equation (Guérin & Dean 2015; Alexandre *et al.* 2025) and centre manifold theory (Mercer & Roberts 1990, 1994; Rosencrans 1997; Marbach & Alim 2019). Many of these methods rely on asymptotic techniques that predict the long-term behaviour as well as incorporate the short-term evolution of the solute concentration (Chang & Santiago 2023).

**Figure 1.**
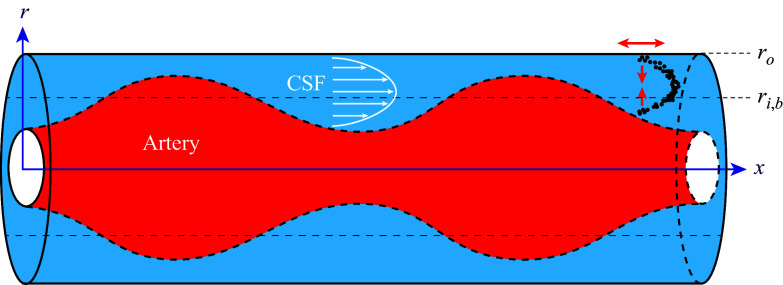
The schematic of the physical system studied in this paper, showing Taylor–Aris dispersion in an annular domain representing a PVS segment. The pulsatile artery is portrayed in red, the surrounding PVS in blue and the CSF flow profile is depicted by white arrows and curves. The solute profile is shown by black dots. The red arrows indicate the shearing caused by the CSF flow profile, which creates radial and axial solute gradients, where advection coupled with radial diffusion enhances axial spreading beyond pure molecular diffusion. The schematic is not drawn to scale.

Motivated by perivascular transport, in this study, we implement asymptotic techniques based on the centre manifold theory, to analyse solute dispersion in annular channels with a spatiotemporally pulsating inner boundary. The centre manifold theory has previously proven effective in quantifying dispersion in geometries with spatiotemporally fluctuating cross-sections (Mercer & Roberts 1990, 1994; Rosencrans 1997; Marbach & Alim 2019). Rooted in nonlinear dynamical systems theory (Wiggins 2003; Carr 2012), the theory projects the slow axial variation of the cross-sectionally averaged solute concentration on a low-dimensional invariant manifold. This yields accurate long-time effective solutions that effectively capture the influence of flow and geometry variations on solute transport (Mercer & Roberts 1990). The main question addressed in this study is how travelling wave-induced deformations of an annular conduit modulate solute dispersion. We derive long-time effective dispersion coefficients, where the influence of the pulsations is separated from the bulk-flow-induced dispersion by additive, phase-averaged correction terms to the effective dispersion equation. The analytical framework developed here is general and can be readily extended to slender annular channels undergoing arbitrary spatiotemporal deformations. Our objective is to elucidate how travelling wave-induced pulsations associated with different brain states, such as during sleep, locomotion and acute physiological conditions, influence solute dispersion in PVSs.

The paper is organised as follows. In § 2 we introduce the governing equations, derive the generalised fluid flow profile induced by pulsations and discuss the implementation of the asymptotic dispersion theory. In § 3 we present our main findings. We begin by deriving a generalised Taylor–Aris dispersion description and obtaining expressions for the long-time effective dispersion coefficients in an annular segment subjected to sinusoidal spatiotemporal deformations of its inner boundary. We next compare the dispersion coefficients with known analytical results for dispersion in annuli with stationary boundaries. We then discuss the dependence of the dispersion coefficients on the wavelength and frequencies of wall deformations in the PVSs. We further demonstrate the applicability of our model using available experimental data. Lastly, we present our concluding remarks in § 4. For clarity, we use the Appendix for presenting validations with numerical simulations of the axisymmetric advection–diffusion equations and detailed derivations supporting the main text.

## 2. Approach

Figure 1 illustrates the physical system under consideration. We seek long-time effective solutions of solute dispersion in a slender annular channel, where the inner boundary undergoes a spatiotemporal deformation described by
(2.1)

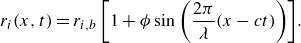

This form of sinusoidal wall deformation wave models a range of physiologically relevant pulsations that the arterial wall of the PVS may exhibit under both normal and acute conditions. Examples include vasomotion due to slow neuronal waves during sleep (Bojarskaite *et al.* 2023), functional hyperaemia, depolarisation events (Mestre *et al.* 2020; Mukherjee *et al.* 2023), neural simulations (Murdock *et al.* 2024) and vasomotion induced by cardiac pulsations (Mestre *et al.* 2018; Kedarasetti, Drew & Costanzo 2022). Here, 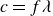 is the wave speed, where 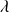 is the wavelength and 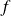 is the frequency, 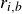 is the base arterial radius and 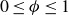 is the normalised amplitude of the wave. The inner radius subjected to the pulsations is bounded in the range 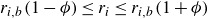 where 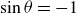 and 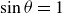 correspond to maximum expansion and maximum contraction of the PVS, respectively, with 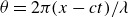 being the wave phase. The outer wall, representing the astrocyte endfeet and surrounding brain parenchyma, is assumed to be rigid and stationary with a radius of 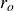 . With a stationary 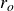 , it is straightforward to obtain an expression of the PVS width or gap 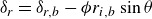 . We use 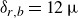 m and 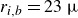 m for most of the study, corresponding to a representative pial PVS (Mestre *et al.* 2018). The exception is § 3.3.4 where we use 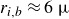 m and 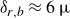 m corresponding to an experimentally measured penetrating PVS (Bojarskaite *et al.* 2023). In the schematic, a representative artery undergoing a sinusoidal deformation is shown in red and the surrounding PVS in blue. The CSF velocity profile in the PVS is indicated in white. This flow profile induces rapid diffusion of the solute concentration in the radial direction, resulting in a nearly uniform profile across the cross-section and varying slowly along the channel length. The radial diffusion and cross-sectional homogenisation are indicated by red arrows.

We next discuss the phase-space spanned by wavelength and frequency of travelling waves in the brain. Recent studies suggest that neuronal oscillations in the brain cortex organise themselves as travelling waves with distinct wavelengths and frequencies (Muller *et al.* 2016; Zhang *et al.* 2018; Liang *et al.* 2021, 2023; Bhattacharya *et al.* 2022). These include delta waves ( 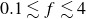 Hz), theta waves ( 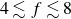 Hz), alpha waves ( 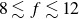 Hz), beta waves ( 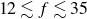 Hz) and gamma waves ( 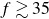 Hz). Spreading depolarisation waves with very low frequencies have typical velocities and wavelengths of 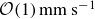 and 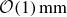 , respectively (Mukherjee *et al.* 2023). Overall, a complex ionic dynamics in the brain during different phases of sleep and wakefulness regulates the generation and propagation of these oscillations (Somjen 2004; Ding *et al.* 2016). Indeed, recent *in vivo* experiments in naturally sleeping and awake mice demonstrate how the vasomotion induced by these waves can dynamically modulate the PVS geometry (Bojarskaite *et al.* 2023). Such vasomotion result from the sensitivity of the arterial lumen to ionic fluctuations, particularly in potassium and calcium concentrations (Knot & Nelson 1998; Farr & David 2011). Similarly, neuronal depolarisation waves induced during seizures, strokes and migraines can induce alterations in the PVS geometry through vasomotion (Mestre *et al.* 2020; Mukherjee *et al.* 2023). Furthermore, recent studies indicate that externally applied neural simulations can drive CSF flow and transport, potentially by inducing arterial vasomotion (Cheng *et al.* 2020; Murdock *et al.* 2024).

Before proceeding further, it is important to clarify the regime where Taylor–Aris dispersion is expected to influence solute transport in the PVSs. In this regime, the shear-induced alteration of the concentration profile results in gradients that are smoothed out by diffusion in the radial direction, operating on a timescale of 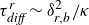 , promoting rapid homogenisation of the concentration field across the cross-section. For Taylor–Aris dispersion to dominate, the cross-sectional homogenisation acts on a faster timescale than axial advection 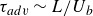 , that is 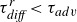 , where 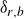 and 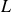 are the PVS width and length, respectively, and 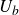 is the average flow speed. Rearranging, the condition yields the bound 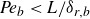 , where 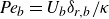 is the Péclet number, describing the competition between bulk flow and diffusion. An additional bound can be derived by noting that the solute molecule travels a relatively large axial extent of 
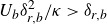
 in the radial diffusion time, which yields 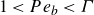 , where 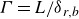 is the aspect ratio of the PVS.

To provide further context, consider the example of solute transport in the PVS involving amyloid beta peptides and tau proteins, both of which are linked with the development of Alzheimer’s disease and other related disorders (Iliff *et al.* 2014; Mukherjee & Tithof 2022). The typical width of pial PVSs (located at the surface of the brain) surrounding the middle cerebral artery in mice is measured to be around 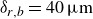 (Mestre *et al.* 2018). Considering an arterial length of 11 mm (Lee *et al.* 2014; Kirst *et al.* 2020), we get an aspect ratio of 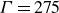 . Now, using typical values of diffusion coefficients of amyloid beta ( 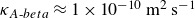 Mukherjee & Tithof 2022) and tau proteins ( 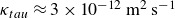 Scholz & Mandelkow 2014), and a measured CSF mean speed of around 20 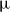 m s^−1^, yields Péclet number values of 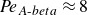 and 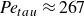 . Both values lie within the range where Taylor dispersion is expected to significantly enhance axial transport. In reality, PVSs, both pial as well as penetrating into the cortex, exhibit significant variations in geometry (Tithof *et al.* 2022). Additionally, other disease-relevant solute molecules like apolipoprotein E (Achariyar *et al.* 2016) and alpha-synuclein (Nedergaard & Goldman 2020), linked with Alzheimer’s and Parkinson’s disease, have low diffusivity, yielding Péclet numbers that lie within the bounds of the Taylor–Aris dispersion regime. These estimates highlight the importance of Taylor–Aris dispersion in the transport of a broad range of solutes and macromolecules in the PVSs. In the presence of spatiotemporal pulsations of the PVS induced by the travelling waves, an additional timescale 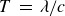 modulates the classical Taylor–Aris dispersion description above, as will be elucidated in this work. Our analytical relies on the comparison of the characteristic transport timescales discussed so far and are summarised in table 1, for ease of reference. A complete list of all parameters used in this paper is summarised in table 3 in Appendix A.

**Table 1.** Characteristic timescales used in this study. The typical magnitudes are obtained by considering an annular conduit of comparable dimensions to a pial PVS segment in murine brain, with baseline PVS width of 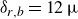 m, length 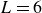 mm and bulk-flow magnitude of 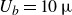 m s^−1^. The wave parameters span physiologically relevant travelling waves observed in the brain. The kinematic viscosity of CSF (water-like) is 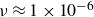 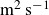 . The solute considered is amyloid beta with diffusion coefficient 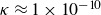 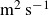 .

Symbol	Expression	Physical meaning	Typical magnitude (pial PVS)
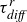	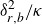	Radial diffusion timescale	1.44 s
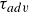	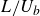	Axial advection timescale	600 s
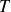	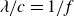	Timescale induced by the wave or wave period	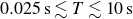
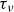	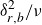	Timescale of radial viscous or momentum diffusion	1.44 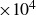 s

### 2.1. Governing equations of solute transport

The transport of a solute with concentration 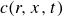 within a long and slender annulus is governed by the advection–diffusion equation as
(2.2)

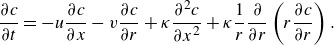

Here, the axial and radial velocities are 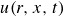 and 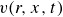 , respectively, and 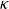 is the diffusion coefficient of the solute. We assume axisymmetry and the coordinate system is cylindrical. We normalise the concentration with a reference maximum value, such that 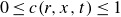 .

Equation (2.2) presents significant challenges in numerically modelling dispersion in slender channels because of the need to resolve the steep radial gradients of the concentration alongside long-range axial variations. As mentioned earlier, fast radial diffusion leads to rapid homogenisation of the concentration across the cross-section, making the cross-sectionally averaged concentration an effective representation of the solute distribution. The relatively slower evolution of the solute along the axial direction can be captured by the reduced one-dimensional model for the average concentration 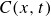 , as
(2.3)

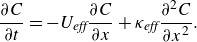




Here, 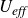 and 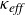 are the effective solute drift and diffusivity, respectively. The shear-induced homogenisation enhances axial dispersion, resulting in an effective diffusion coefficient that exceeds molecular diffusivity ( 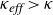 ). Equation (2.3) conveniently bridges the gap between theory and experiments where solute spreading is typically measured along the channel. The resulting reduced-order model also provides a simplified, yet physiologically accurate, representation of solute transport that is also computationally efficient. The solute drift represents the time rate of change of the first moment (or centroid) of the solute bolus, as defined by 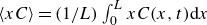 . In the absence of pulsations, the effective solute drift is equal to the cross-sectionally averaged, or bulk axial velocity 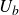 . However, when the flow is unsteady or exhibits substantial axial variations, such as those induced by a moving boundary, 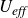 can deviate from 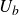 (Marbach & Alim 2019). Our overall goal is to find the effective dispersion coefficients, 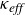 and 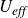 , in a long and slender annular channel that is subjected to spatiotemporal fluctuations corresponding to travelling waves in the brain.

### 2.2. Fluid flow profile

We consider an annular segment undergoing spatiotemporal pulsations of its inner radius of the form shown in (2.1). Under the lubrication approximation and in the limit of negligible wave-induced inertia, the velocity profile can be treated as quasi-Poiseuille, driven by a spatiotemporally varying axial pressure gradient 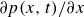 that is induced by the wall pulsations. The flow profile is given by (White 2006)
(2.4)

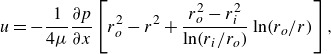

where 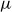 is the coefficient of dynamic viscosity of the fluid. Equation (2.4) has the familiar form of the Poiseuille flow profile which is valid under the approximations described below. A detailed derivation of this flow profile is provided in Appendix B, where we non-dimensionalise the axisymmetric momentum equation using a length scale of 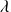 in the axial direction, baseline PVS width 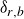 in the radial direction and a timescale of wave period 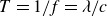 . Doing so, we obtain two non-dimensional parameters, the wavenumber, defined as 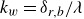 and the wave-induced Reynolds number 
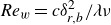
 .

The wavenumber 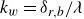 quantifies the inner wall curvature of the annular conduit subjected to the pulsatile deformation. For PVS geometries subjected to pulsations, we find 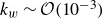 , enabling the lubrication approximation and implying 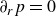 . Additionally, the deformation amplitude is weak 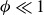 , which together with 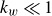 ensures that the instantaneous wall curvature remains small at all times. Here, 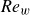 is the ratio of the timescale of viscous diffusion 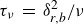 and the wave passage time over one wavelength 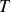 . For the physiological wave parameters and PVS dimensions considered here, we find that 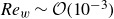 , indicating negligible wave-induced inertia. Indeed, 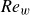 is related to the Womersley number, defined using the mean PVS width, 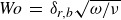 , as 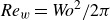 , and past studies have indicated that 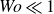 for CSF flow through the spinal canal and PVSs (Sánchez *et al.* 2018; Kelley & Thomas 2023). Under the limits of 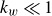 , 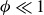 and 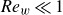 , that is small wall curvature and negligible wave-induced inertia, the quasi-Poiseuille flow profile (2.4) is recovered from (B7) (Appendix B).

Additionally, when the ratio of the annular thickness to the inner radius is substantially smaller than unity, that is 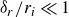 , the flow profile above can be simplified into a parabolic flow profile by expanding the logarithmic term (White 2006)
(2.5)

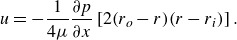

The thin annulus approximation is appropriate for PVSs, as the annular thickness is typically small. Specifically, the annular cross-sectional area ratio of the PVS to the artery, 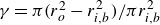 , has an upper limit of 1.4 for pial PVS in mice (Tithof *et al.* 2022), which yields an upper limit of 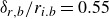 and supports the use of the thin annulus assumption. The pressure gradient term can be substituted in (2.5) by rearranging the expression with the cross-sectionally averaged velocity 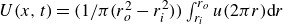 , as
(2.6)

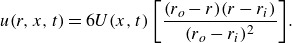




The flow profile (2.6) is parabolic with a maximum velocity of 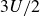 at the centreline of the annulus, 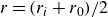 . The flow is governed by Dirichlet boundary conditions at the walls. The spatiotemporal contractions in the inner wall imply that a radial component to the velocity must be present. The boundary conditions at the outer wall is 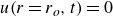 and 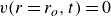 . Similarly, at the inner wall, we have 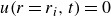 and 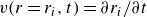 . Using the continuity equation 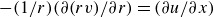 and applying the boundary conditions yields the following expression for the radial component of the velocity:
(2.7)

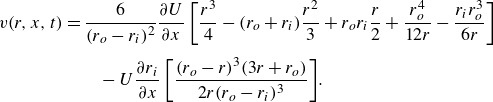

The expressions of both the radial and axial components of the velocity depend on the cross-sectionally averaged velocity 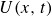 . Using conservation of mass on a control volume enveloping the annular segment, it can be shown that the cross-sectionally averaged fluid velocity 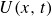 relates to the spatiotemporally varying cross-section by (Ottesen 2003; Sherwin *et al.* 2003)
(2.8)

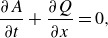

where 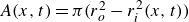 is the cross-sectional area, and 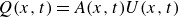 is the volume flow rate. An expression for 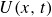 can be obtained by integrating (2.8), 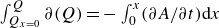 , and considering an inlet condition for the flow rate 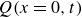 . The expression of 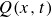 obtained after such integration is
(2.9)

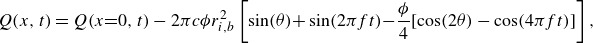

where 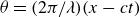 . We assume that the volumetric flow rate at the inlet of the annular segment comprises of a steady and a temporally fluctuating component
(2.10)

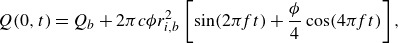

where 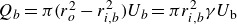 is the time-invariant part of the flow rate at the inlet and 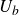 is the bulk-flow speed at the inlet. In the context of PVSs, this expression of flow rate at the inlet can be interpreted as the average volume flow rate of CSF in the subarachnoid space or pial PVSs branching into the cortex, before entering the PVS segment under investigation (Mestre *et al.* 2018; Kedarasetti *et al.* 2022). Equation (2.9) is directly obtained from integrating the one-dimensional mass conservation (2.8), which assumes incompressibility and impermeable, non-leaky walls. It is insightful to expand (2.9) in orders of the wave amplitude to yield
(2.11)

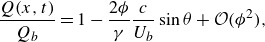

which is a convenient leading-order reduction with 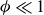 , where 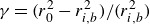 is the PVS area ratio. The modulation to the bulk-flow rate is controlled by the dimensionless combination 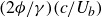 , which couples the deformation amplitude, and the wave-to-bulk speed ratio. In our study, 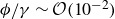 and the modulation is small when 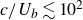 . For small modulation, the reduction implies that the phase average of the flow rate over one period is equal to the bulk-flow rate at the inlet, 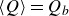 . In the small modulation regime, the flow rate reaches a maximum value of 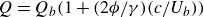 when 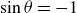 , corresponding to PVS expansion, and a minimum value of 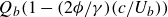 when 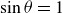 , corresponding to PVS contraction. However, for the physiological travelling waves we study, the wave speeds corresponding to travelling waves in the brain are much faster than the bulk-flow speed of CSF in the PVS with 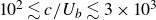 . This implies strong oscillatory modulation to the flow rate in expansion and contraction events, and the 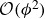 term becomes important. Accordingly, we employ (2.9) for all cases we study.

The cross-sectionally averaged flow velocity can be then determined by dividing (2.9) by the area to yield
(2.12)

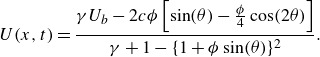

For small modulation, (2.12) can be expanded in orders of the wave amplitude as 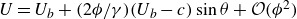 , which we will find to be a convenient simplification with 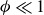 , and implies that the phase average of the velocity is equal to the bulk fluid velocity, 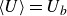 . Also, the bulk flow is unaffected by the pulsations when 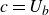 , or when the wave speed is equal in magnitude to the bulk-flow speed with the wave propagating downstream. At leading orders, the cross-sectionally averaged velocity reaches two peaks of magnitude 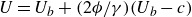 when 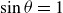 , corresponding to PVS contraction, and 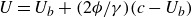 , when 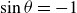 , corresponding to PVS expansion. The difference between the peaks corresponding to expansion and contraction is 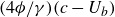 , implying that, for 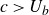 , PVS expansions results in faster mean axial flows than contractions, which holds for physiological travelling waves in the brain. The leading-order simplification is, however, only true when the modulation 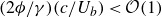 . Thus, similar to the flow rate, we implement the full equation of 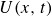 (2.12) with all orders of 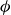 to accommodate cases where the modulation is strong.

**Figure 2.**
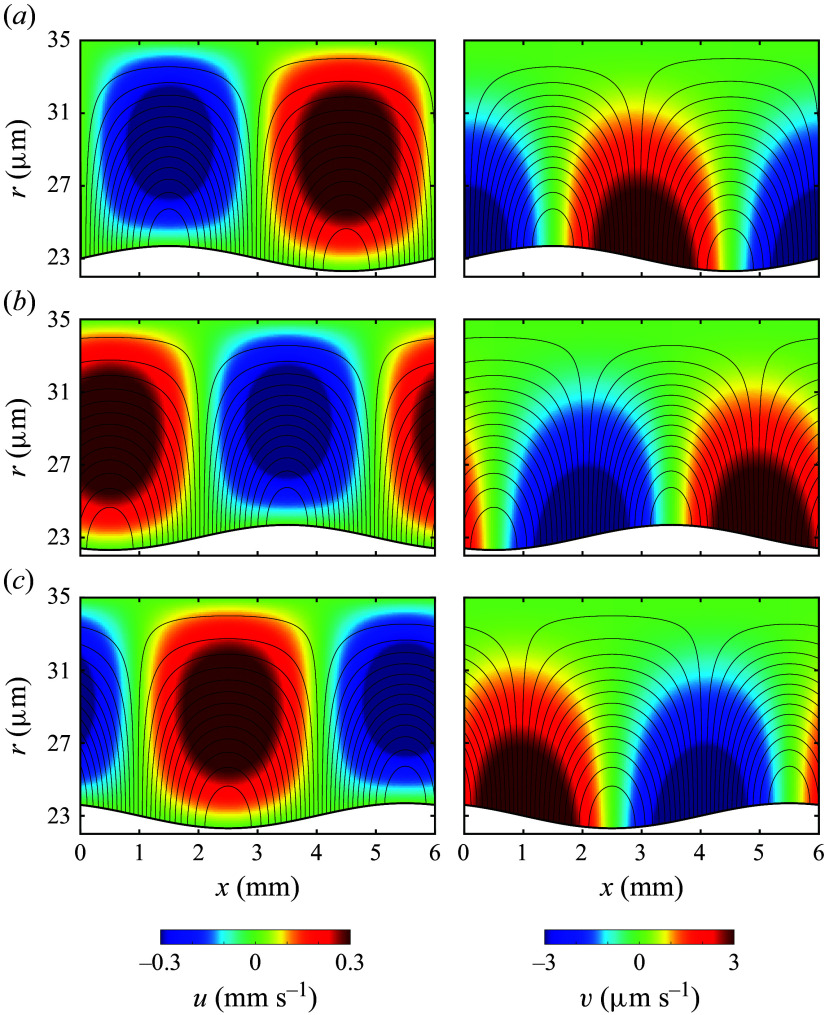
The colour contours of the magnitude of the axial and radial components of the fluid velocity at three different instances of time. Here, 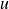 is shown on the left and 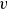 on the right of each panel. Time increases downward with: 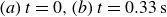 and 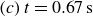 , corresponding to three characteristic wave phases 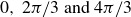 , of the wall deformation wave, respectively. The streamlines are overlaid on top of the colour contours. The radial axis is exaggerated for the ease of visualisation ( 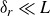 ). Relevant parameters: 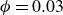 , 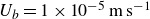 , 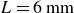 , 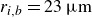 , 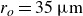 .

We plot the spatiotemporal dynamics of the flow profile due to the peristaltic wall wave in figure 2, where the colour contours of the components of the velocity field 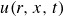 and 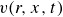 are plotted at certain instances of time. The radial axis in the figure has been exaggerated for visualisation. The times chosen for the visualisation are at phases 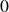 , 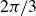 and 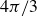 , respectively. Each panel shows 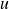 in the left and 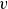 in the right for the chosen times. Figure 2(*a*) shows the colour contours of 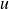 and 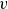 respectively when 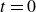 . The PVS expansion induces axial flow downstream (shown in red) while PVS contraction pushes fluid axially upstream. The maximum magnitude of the axial component generated is approximately 4 mm s^−1^ in either direction for this case. The radial component 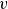 is induced on either sides of the maximum expansion or contraction, aligning with locations where the axial flow is weak. Figures 2(*b*) and 2(*c*) show the colour contours of the axial and radial components at the phases 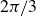 and 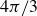 , respectively, and show expected trends of 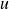 and 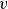 , as discussed above.

Figure 2 implies that the axial component of the velocity induced by the wave is two orders of magnitude greater than the radial component. This results from the large aspect ratio of the problem with the wavelength considerably exceeding the PVS width, 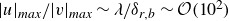 . The expression of the axial component of the fluid flow 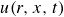 in (2.6) predicts a time-varying parabolic flow profile that is largest along the centre. The expression of the radial component 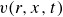 (2.7) consists of a first term that is proportional to 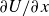 and accounts for axial inhomogeneity driving the radial velocity. The second term in 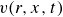 is proportional to 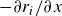 or the peristaltic pumping. The radial velocity thus changes sign either side of a maximum expansion or contraction. We have 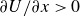 and 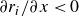 on the upstream side of a PVS expansion, resulting in inward flow, for instance, 0 mm 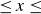 2 mm, in figure 2(*c*). Similarly, 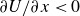 and 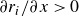 on the downstream side of the expansion resulting in radially outward flow, for instance 3 mm 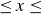 5 mm, in figure 2(*c*). The shear-driven advection of the solute induced by this fluid flow profile coupled with fast radial diffusion leads to enhanced Taylor–Aris dispersion of the solute concentration, as elaborated in the subsequent sections of the paper.

### 2.3. Asymptotic dispersion equation

We seek to find an asymptotic dispersion equation that separates the fast timescale of radial diffusion and the relatively slower timescale of axial advection. We use a straightforward perturbation expansion to obtain the reduced set of dispersion equations (Marbach & Alim 2019; Nayfeh 2024). We first transform (2.2) into its non-dimensional form to enable asymptotic expansion using characteristic scales such as
(2.13)

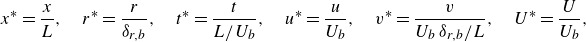

where 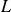 is the domain length, 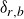 is the mean annular thickness (PVS width) and 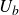 is the bulk-flow speed. The non-dimensional form of (2.2) is
(2.14)

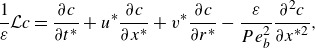

where 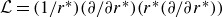 is the linear operator, 
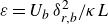
 is a small parameter and 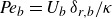 is the base Péclet number. The small parameter 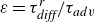 is the ratio of the radial diffusion timescale 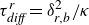 and the axial advection timescale 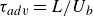 .

As discussed earlier, in the Taylor dispersion regime, 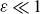 . This approach of separating the fast and slow timescales is derived from the dynamical systems theory of centre manifolds (Carr & Muncaster 1983; Wiggins 2003). The centre manifold of a dynamical system is a type of ‘invariant manifold’ that can be obtained by linearising the dynamics about a reference point or trajectory and conducting an eigen vector analysis. The three invariant subspaces that the linearised dynamics of the system can evolve in are stable, unstable or centre. The dynamics typically evolves rapidly along the stable and unstable directions, and slowly along the centre direction, which describes the long-term behaviour of the dynamics. This theoretical framework has been found to be particularly useful to reduce the dimensions of physical systems that manifest a dynamics evolving on a fast and a slow timescale, such as Taylor dispersion (Roberts 1988; Mercer & Roberts 1990, 1994; Marbach & Alim 2019).

Following Marbach & Alim (2019), we seek a straightforward perturbation expansion in the small parameter 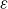



(2.15)

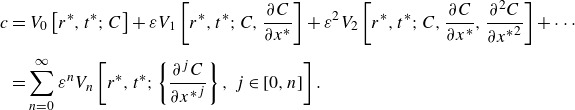

We also look for the expansion of the cross-sectionally averaged concentration 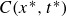 as
(2.16)

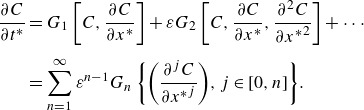

We can now substitute (2.15)–(2.16) in (2.14) to obtain the successive orders of 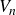 and 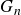



(2.17)

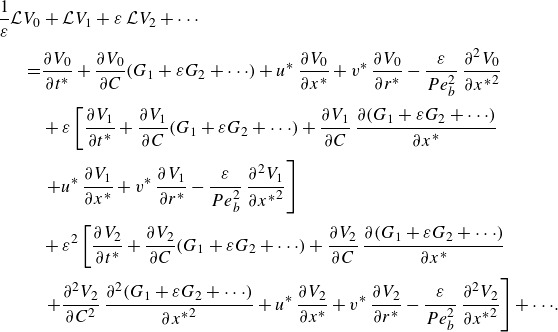




In the above, chain rule has been implemented for the differentiation of 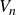 . Grouping the terms by order of 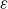 leads to the system of equations given by
(2.18)

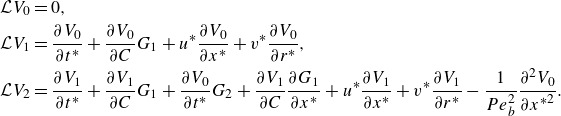

Here, we have included only two orders of the perturbation expansion, which we have found to be sufficient to resolve the full system. It is important to note that the system of equations in (2.18) can also be obtained by applying a Fourier transform to (2.14) with respect to the longitudinal spatial variable 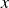 and using the asymptotic expansions for 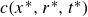 and 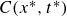 . This has been implemented by Mercer & Roberts (1990, 1994). All the orders of the perturbation expansion must also satisfy the boundary condition for the solute concentration. We have implemented a no-flux boundary condition for 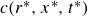 , such that 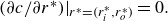 where 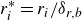 and 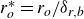 denote the normalised inner and outer radii, respectively. This yields the boundary condition
(2.19)

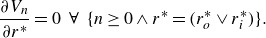




Finally, closing equations can be derived from the relation between the cross-sectionally averaged concentration field and the solute concentration, by 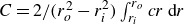 . Using non-dimensional parameter 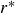 instead of 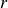 and expanding 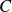 as per (2.15), yields
(2.20)

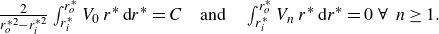




## 3. Results and discussion

### 3.1. Effective dispersion coefficients

We seek to obtain expressions for the effective dispersion coefficients 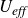 and 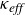 in (2.3). Upon solving the system of equations in (2.18), we get
(3.1)

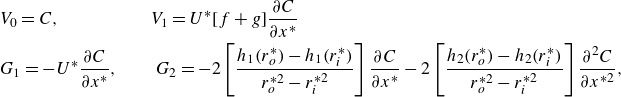

where the expressions for 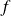 , 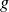 , 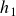 and 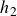 are detailed in Appendix D. Substituting in (2.16) yields
(3.2)

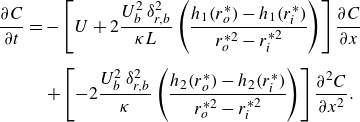

Comparing (2.3) and (3.2), we get the formulations of the effective dispersion coefficients. Specifically, 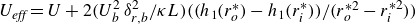 and 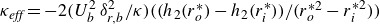 . We simplify and reorganise the coefficients to yield further physical insights in relation to the pulsatile inner radius 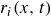 and the cross-sectionally averaged fluid flow velocity 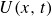 . The details of the simplification and the derivation are presented in Appendix E. The simplified effective dispersion coefficients are 
(3.3)

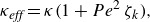



(3.4)

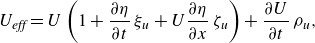

where 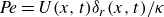 is the local spatiotemporally varying Péclet number. The prefactors 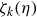 , 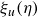 , 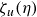 , 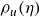 are log-polynomial functions of 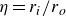 , the ratio of the inner and outer radii. Their full expressions are provided in the Appendix D. The expressions of the dispersion coefficients presented in (3.3)–(3.4) are cast in a way that is implementable for arbitrary forms of radial pulsations 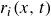 and mean axial flow profile 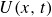 .

Equation (3.3) implies that the effective diffusivity gets enhanced compared with its base value due to the shear-induced cross-sectional homogenisation and enhanced axial spread. The enhancement scales as 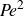 multiplied by a geometric factor 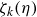 . The form matches the typical expectation of diffusion coefficient enhancement in Taylor–Aris dispersion. It is interesting to note, however, that the enhancement is a function of a spatiotemporally varying ‘local’ Péclet number, implying that local variations in the geometry and the fluid velocity induced by pulsations, modulate the effective diffusivity.

The effective solute drift, given by (3.4), is similarly enhanced compared with the cross-sectionally averaged velocity 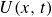 . The enhancement involves: (i) a correction due to the pulsatile wall motion, 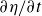 , with local dilation and contractions leading to local acceleration of the solute, (ii) a correction due to the spatial wall gradients, 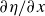 , capturing entrainment of the solute in regions of varying cross-section and (iii) a correction due to the time-varying mean flow speed 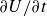 , indicating the response of solute transport to acceleration/deceleration of the mean flow.

The effective dispersion coefficients derived in (3.3)–(3.4) are instantaneous and depend on the pulsatile geometry 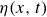 and the mean flow speed 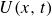 . This dependence is linked to the pulsatile timescale 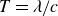 , that enters parametrically through the spatiotemporally varying flow speed, 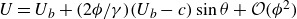 , and geometry, 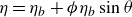 . When averaged over a cycle, 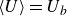 , at leading orders, and 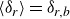 ; however, the pulsations produce additive enhancements to the long-time effective dispersion coefficients, as will be discussed below. To quantify the effect of the pulsations, we will find analysing the mean enhancements, given by 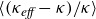 and 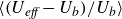 , to be useful. For determining the mean enhancement values, we calculate the phase average of the dispersion coefficients over one period of the wave 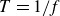 and over the domain length 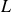 .

**Figure 3.**
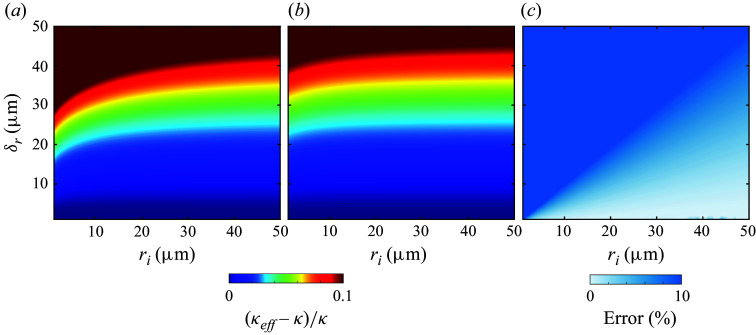
The colour contours of the diffusivity enhancement as a function of radius and annular thickness in a domain without pulsations and of length 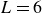 mm. The molecular diffusivity of the solute is 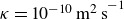 and the bulk-flow velocity 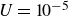 m s^−1^. (*a*) Diffusivity enhancement from the analytical solution provided by Aris (Aris 1959). (*b*) Diffusivity enhancement predicted by (3.4). (*c*) The normalised percentage error between the diffusivity enhancements in (*a*) and (*b*).

### 3.2. Taylor–Aris dispersion in a segment of PVS without pulsations

Before quantifying dispersion in a slender annulus with a pulsatile wall, we solve the classical problem of dispersion in a slender annulus with stationary walls, for which analytical solutions are available (Aris 1959). This serves as a validation of the effective dispersion coefficients derived in the prior section. Additionally, this provides a benchmark for comparison when pulsatile walls are imposed. The validation involves comparing the mean enhancement in the effective diffusion coefficient of a solute, 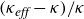 , between our asymptotic analysis, given by (3.3), and Aris’s analytical solution (presented in Appendix F).

The length of the annular segment is prescribed as 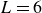 mm, which is comparable to unbranched segments of arteries measured in murine brain (Blinder *et al.* 2010). Since the geometry is not undergoing pulsations, 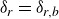 and 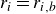 . We plot 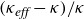 for various annular thicknesses 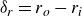 and inner radii 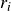 for a solute amyloid beta with molecular diffusivity 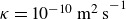 . It is important to note that, for this non-pulsatile case, the solute drift is simply equal to the cross-sectionally averaged flow profile, 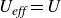 . This can be verified from (3.4), where in the absence of wall pulsations, 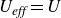 . We impose the parabolic flow profile in (2.6) with a mean of 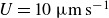 , which is comparable to flow speeds measured *in vivo* (Mestre *et al.* 2018).

Figure 3 shows the comparison between the analytical solution of the dispersion coefficient in panel (*a*) and the effective dispersion coefficient given by (3.3) plotted in panel (*b*). The analytical solution by Aris (1959) is presented in Appendix F. The colour contours of the diffusivity enhancement is plotted as a function of 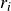 and 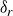 in figure 3(*a*–*b*) in the range 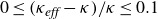 . Overall, the shear-induced relative dispersion enhancement is always greater than zero and reaches a maximum value for lowest 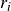 and highest 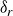 values considered. Comparing the analytical solution in (a) and the asymptotic solution in (b), we find similar enhancement with modest deviations for 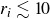 and 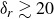 .

To further understand the source of this deviation, we plot the relative percentage error between the asymptotic and the analytical solution in figure 3(*c*). We find that our model performs particularly well when 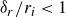 . Indeed, this condition was an original assumption in our model allowing us to approximate the flow profile as parabolic. As 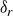 decreases, the error between the model and Aris’s solution approaches zero, confirming the validity of the model in thin and slender annular geometries. However, when 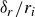 increases, the error becomes more significant due to deviations from the parabolic flow approximation, which neglects the logarithmic corrections inherent in the Poiseuille profile for annular flow. The diagonal in figure 3(*c*) represents 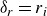 , and separates the large and small error regimes. Overall, the dispersion coefficient agrees reasonably well with the analytical solution with a modest 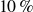 error when the annular thickness exceeds the inner radius thickness.

**Figure 4.**
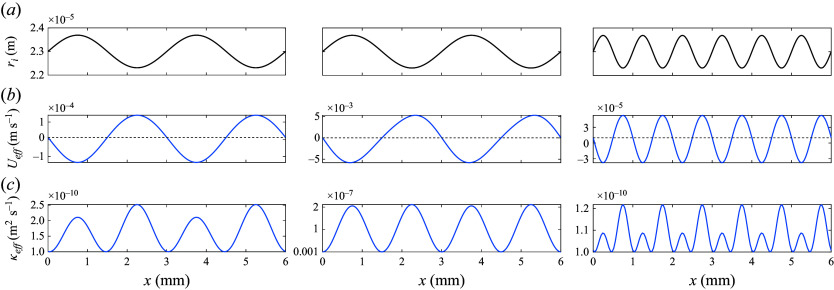
Spatial fluctuations of the inner radius (top), effective drift (middle) and the effective diffusivity (bottom) at select time instances for a wall deformation wave for varying frequencies and wavelengths incident on an annular segment representing a pial PVS. Panels show (*a*) 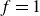 Hz and 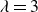 mm, (*b*) 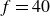 Hz and 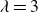 mm and (*c*) 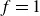 Hz and 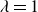 mm. Relevant parameters: 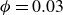 , 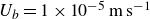 , 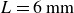 , 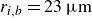 , 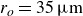 .

### 3.3. Effective dispersion in a segment of PVS

We next quantify the behaviour of the dispersion coefficients in an annular PVS segment with pulsatile walls. The bulk-flow speed at the inlet is prescribed at 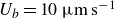 and the solute molecule studied is amyloid beta with 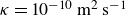 . The base value of the arterial radius is chosen as 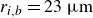 , and the base annular PVS width is 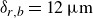 , resulting in an area ratio of 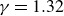 , representing a typical pial PVS dimension in mice. The amplitude of the wall oscillation is prescribed at 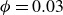 . The PVS dimensions and value of the wave amplitude are consistent with experimental measurements in mice (Mestre *et al.* 2018; Tithof *et al.* 2022; Bojarskaite *et al.* 2023), and past numerical studies of perivascular flows (Mestre *et al.* 2018; Carr *et al.* 2021; Kedarasetti *et al.* 2022; Bojarskaite *et al.* 2023).

Since no analytical solutions for dispersion coefficients exist in the presence of pulsations, we compare our expressions with those obtained from a finite-difference simulations of the solute evolution. The dispersion coefficients from the simulations are obtained by calculating the moments of the solute concentration and the mean square displacement (MSD). The validation, presented in Appendix C, shows reasonable agreement between the dispersion coefficients obtained from simulations and from our analytical expressions.

We first examine the spatial variation of the inner radius and the dispersion coefficients for an annular segment at different frequencies and wavelengths of the wall wave at a fixed time 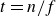 , where 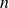 is an integer, corresponding to a phase of 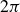 . Each panel in figure 4 shows the spatial variation of 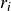 , 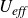 and 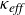 from top to bottom, respectively. Figure 4(*a*) shows the case of 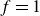 Hz and 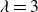 mm. The sinusoidal fluctuation of the inner radius corresponding to (2.1) for 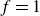 Hz and 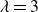 mm is shown in the top plot. The effective solute drift undergoes smooth sinusoidal variations in space, with maxima and minima at 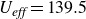 and 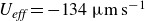 , respectively. The dotted line indicates 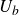 . The diffusion coefficient exhibits a bimodal sinusoidal variation with peaks of 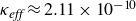 and 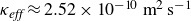 . Localised expansion of PVS, indicated by troughs in the radial deformation wave, correspond to enhancement in drift and diffusivity. The localised increase in the PVS area leads to forward flow downstream and enhancement in the diffusivity of the solute molecules, which scales as 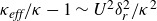 . The peaks in the radial wave correspond to PVS contractions that result in backflow, as evident from the negative value of the drift. The magnitude of the backflow is smaller than the downstream axial flow and produces smaller peaks in the diffusivity.

Figure 4(*b*) shows the results for 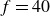 Hz and 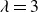 mm. Increasing the frequency yields stronger upstream and downstream drifts with values of 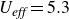 and 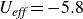 mm s^−1^ during expansion and contraction, respectively. The diffusion coefficient exhibits oscillations in the range 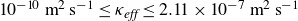 . Compared with panel (*a*), the contraction events produce peaks in diffusivity that are comparable to the expansion events, reflecting the symmetry in the induced forward and backward flow magnitude.

The results for 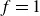 Hz and 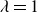 mm are shown in figure 4(*c*). The troughs in the inner radius fluctuations, corresponding to PVS expansion, again coincide with the peaks in solute drift and diffusivity. The contractions of the PVS correspond to troughs in the effective drift and reduced peaks in the diffusivity. The pulsations for this slower wave speed induce a relatively lower fluid flow compared with the previous two cases, as evident from the troughs and crests of the drift at 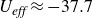 and 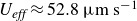 , respectively. As a consequence, the peaks of the diffusivity receive negligible enhancement from the baseline value of 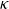 .

#### The effect of varying frequency 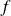 and wavelength 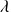 of the travelling pulsations

3.3.1.

We next analyse the effect of varying the frequency and wavelength of the radial deformation wave on the dispersion coefficients over a range of frequencies, 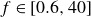 Hz, and wavelengths, 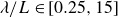 . These frequency and wavelength bounds are physiologically informed to encompass travelling waves in the brain such as slow waves during sleep, cardiac pulsations and neural simulations, as discussed earlier. The amplitude of the wave is held constant at 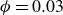 .

**Figure 5.**
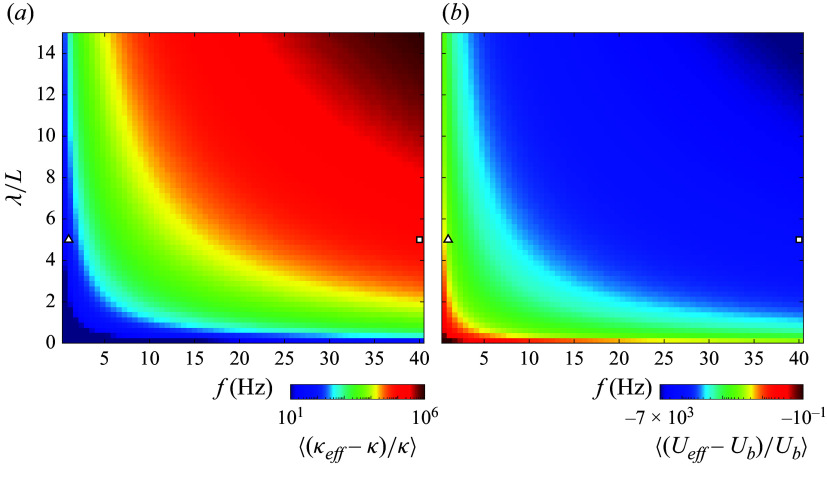
The colour contours of mean enhancements of the Taylor–Aris dispersion coefficients for a solute in a pulsatile annular segment representing a pial PVS. The plot is over the range of physiologically relevant frequencies 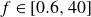 and normalised wavelengths 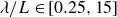 of travelling waves in the brain. The amplitude is held constant at 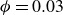 . Contours of (*a*) mean diffusivity enhancement and (*b*) mean drift enhancement across the specified frequency and wavelength ranges. The white triangular and square markers are representative low- and high-frequency cases that are simulated in figure 9. The colours scale logarithmically in both the panels. Relevant parameters: 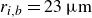 , 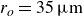 , 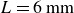 , 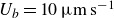 , 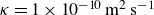 , 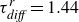 s and 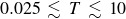 .

We plot the spatiotemporally mean values of enhancements of the dispersion coefficients as a function of the wavelength and frequency in figure 5. The averaging was performed over one wave phase over 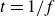 and the entire domain length, 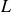 . We have found that our results do not substantially change upon changing 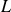 , so the *Y*-axis is expressed in 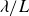 for generality.

The mean enhancement in diffusivity is shown in figure 5(*a*). The data are represented as colours that scale logarithmically with red and blue denoting large and small magnitudes, respectively. Diffusivity increases with both wavelength and frequency, reaching a maximum value of 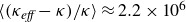 for 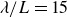 and 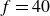 Hz. The mean enhancement in solute drift 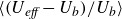 is shown in figure 5(*b*). The colours indicate qualitatively similar enhancement behaviour as the diffusion coefficient, which increases in magnitude for larger wavelength and frequencies. The mean enhancement in the drift is negative for the frequencies and wavelengths studied, indicating a net upstream movement from right to left of the domain on average. The maximum enhancement in drift is 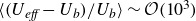 .

#### 3.3.2. Long-time effective dispersion coefficients

The mean enhancements in the dispersion coefficients, 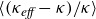 and 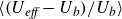 , obtained by phase averaging (3.3)–(3.4), represent the long-time effective dispersion coefficients of the solute. In this regime, the observation time satisfies 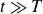 , such that many pulsation cycles have elapsed and the wave-induced contributions are phase averaged. Taking advantage of the fact that the phase average of a sinusoidal wave is zero, and using Taylor series expansions for small magnitude of pulsations, that is 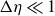 , the mean diffusivity enhancement can be written in terms of the bulk Péclet number 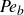 and the wave Péclet number 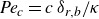 , as
(3.5)

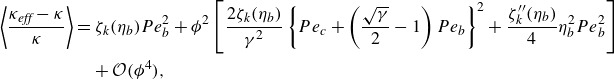

where 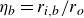 and 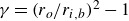 is the area ratio, as before. Here, 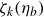 is the form factor in the diffusion coefficient expression shown by (3.3). The wave Péclet number, 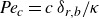 , is a key dimensionless parameter which quantifies the competition between the timescales of radial diffusion 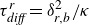 and the wave-induced transport across a length equal to the annular width 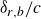 . Indeed, the ratio of the wave-to-bulk Péclet numbers satisfies 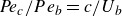 , and directly compares the transport induced by the travelling wave with that induced by the bulk flow. The details of the derivation of the mean diffusivity enhancement are provided in Appendix E.1. We find that only even orders of 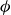 are retained because the odd powers vanish upon phase averaging. Because measured pulsation amplitudes in the brain are small, 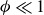 , higher-order terms can be neglected, indicating that the mean diffusivity enhancement scales as the square of the wave amplitude 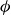 .

Equation (3.5) decomposes the mean enhancement in diffusivity over the baseline 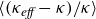 , into enhancements due to the bulk flow and additive wave-induced correction terms. The first term in (3.5), 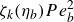 , indicates the diffusivity enhancement due to the bulk flow 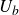 , in the absence of the wave. This enhancement results from the shear induced by the bulk parabolic flow profile in the annular domain. The first term vanishes for 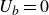 , or no bulk flow. The wall pulsations result in two additive, phase-averaged correction terms to the long-time effective diffusivity.

The first correction term, 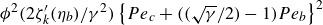 , is a positive contribution to the dispersion arising from the back-and-forth solute motion induced by the oscillatory axial flow. This phenomena is called ‘shuttle dispersion’ (Watson 1983; Marbach & Alim 2019), where the pulsations induce oscillatory axial flows that advect the solute molecule forward and backward, inducing greater axial spread (Schmidt *et al.* 2005). Shuttle dispersion increases for larger values of 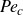 , or when the wave transports solute across the gap faster than diffusion. Indeed, shuttle dispersion is non-zero even without any bulk flow, 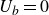 but increases with bulk flow due to the corresponding increment in 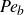 . The shuttle dispersion term vanishes at the critical speed ratio of
(3.6)

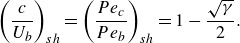

Below this limit, the shuttle dispersion is negligible, while above this limit, shuttle dispersion contributes significantly to the Taylor dispersion of the solute. For 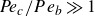 , which correspond to travelling waves in the brain, the diffusivity scales as 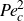 or 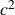 . Since, 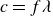 , this implies that for constant 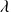 , diffusivity increases quadratically with frequency, and *vice versa*. The quadratic dependence on 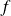 and 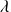 agrees with the nature of the contours of the mean diffusivity enhancement plotted against the frequency and wavelength in figure 5(*a*).

The second correction term, 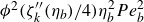 , is independent of the wave speed. Indeed, this term is negative because 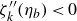 for a thin annulus. This negative contribution to the dispersion is called ‘entropic slowdown’ resulting from the solute molecules being transiently trapped in the cavities and constrictions created by wall pulsations. Entropic slowdown is independent of the wave speed 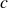 but depends on the form factor 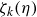 . The ratio of the entropic slowdown correction to the first term is 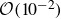 , which implies that there is a very minor influence of the slowdown in annular geometries corresponding to PVSs. The effect is localised in the confinements created by pulsations and does not substantially influence the overall dispersion enhancement. Additionally, entropic slowdown is only significant below the critical wave speed at which the shuttle dispersion vanishes.

Indeed, a critical ratio of the wave-to-bulk Péclet numbers can be obtained by equating the second-order wave-induced correction term in (3.5) to zero. At this speed, the contribution of the pulsations, both shuttle dispersion and entropic slowdown, to the enhancement in diffusivity vanishes. This critical ratio is
(3.7)

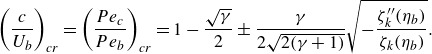




At this critical limit, the shuttle dispersion is fully nullified by the entropic slowdown. For 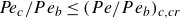 , shuttle dispersion is negligible, and dominated by entropic slowdown, which is independent of the wave speed. It is important to note that the expression in (3.7) can be positive or negative, indicating that the wave speed can be in the same direction as 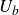 , as in the present case, or opposite.

It is interesting to note that the long-time effective diffusivity given by (3.5) is not always ‘asymptotic’ in the classical sense of Taylor–Aris dispersion, because the wave-induced timescale 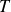 can be comparable to or even smaller than the radial diffusion timescale 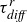 , in certain regimes (table 1). This is particularly true at high wave speeds, where with 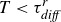 , the instantaneous cross-sectional homogenisation of the solute is not guaranteed in a single pulsation period. A direct relationship, in terms of wave Péclet number, can be obtained when the radial diffusion timescale equals the wave-induced timescale 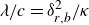 , which yields 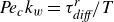 , where 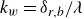 is the wavenumber, as described in § 2.2. Below this bound on the wave Péclet number, the system can be considered asymptotic in the classic Taylor–Aris dispersion sense when 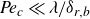 . Expressing the bound in terms of the PVS area ratio 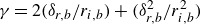 , yields
(3.8)

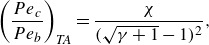

where 
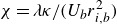
 is a wavelength transport parameter related to the ratio of the timescale of advection over one wavelength 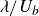 to the diffusion timescale over the arterial radius 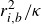 . For pial PVS parameters in mice and a wavelength equal to 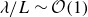 , we find that 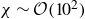 .

Equation (3.8) provides a threshold, below which, the cross-sectional homogenisation of the solute is completed at every wave period, 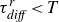 . In this regime, the dispersion coefficients can be considered ‘asymptotic’ in the classical Taylor–Aris dispersion sense with 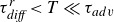 . However, even with 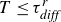 , the combined effect of the oscillatory shear and the radial diffusion, produces net axial spreading that grows linearly with time upon phase averaging. The oscillatory shear repeatedly redistributes the solute while the diffusion acts cumulatively over many cycles. Over long times 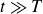 , the average contribution of these mechanisms corresponds to two correction terms, which are additive, to the base Taylor–Aris dispersion due to the bulk flow in (3.5).

Equation (3.5) thus represents the long-time, effective, phase-averaged dispersion coefficient by averaging over many pulsation cycles. These long-time dispersion coefficients are asymptotic or pre-asymptotic in relation to transverse homogenisation, depending on whether 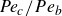 lies below or above the bound in (3.8). In both regimes, using finite-difference simulations of (2.2) we find that the oscillatory MSD exhibits linear growth in time and yields effective diffusivity values consistent with (3.5). A representative validation in the asymptotic regime is presented in Appendix C. In the absence of wall pulsations, the dispersion coefficients reduce to the classical Taylor–Aris results and show good agreement with the Aris solution, as elaborated in § 3.2. Thus, overall, the pulsation-induced effect is now separated from the bulk-flow-induced dispersion by the two additive correction terms which correspond to shuttle dispersion and entropic slowdown.

**Figure 6.**
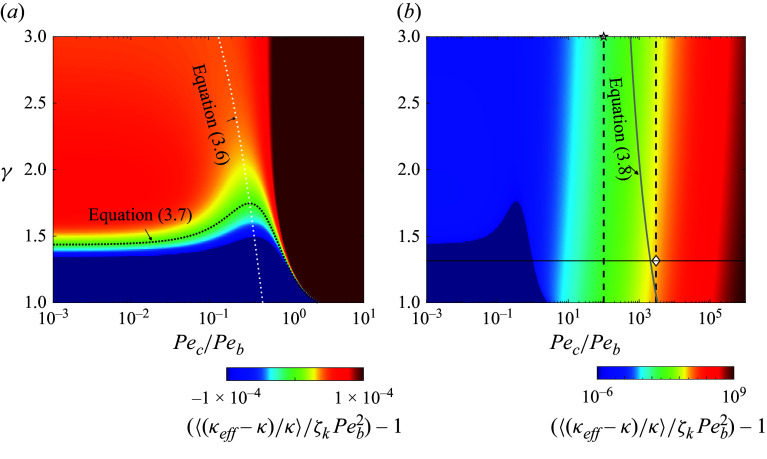
The colour contours of the mean enhancement in diffusivity 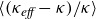 , normalised by its value in the absence of pulsations 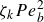 , plotted as a function of area ratio and wave-to-bulk Péclet number ratio following (3.5). (*a*) For 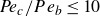 . The white dotted line is the wave-to-bulk Péclet number ratio at which shuttle dispersion vanishes 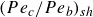 (3.6). The black dotted line is the critical wave-to-bulk Péclet number ratio 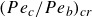 at which the wave does not contribute to any enhancement ((3.7)). (*b*) For 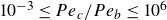 , with colours scaling logarithmically. The black vertical dashed lines denote a representative range of physiological travelling waves in the brain 1 mm s^−1^ 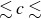 30 mm s^−1^ Liang *et al.* (2023). The grey solid line is computed using (3.8), with 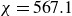 , corresponding to a representative PVS, and provides the bound of 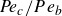 beyond which 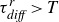 . The black solid horizontal line represents the area ratio 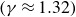 corresponding to a representative pial PVS geometry used in the majority of the study, and the white diamond marker represents waves with constant speed of 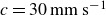 shown in figure 8. The white star indicates 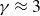 and 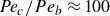 corresponding to the penetrating PVS results in table 2.

Figure 6 shows the colour map of the magnitude of mean enhancements in diffusivity, induced by the wave, as a function of the wave-to-bulk Péclet number ratio and area ratio, based on the analytical expression in (3.5). In order to quantify the effect of the wave, we have normalised the mean enhancement in diffusivity 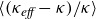 by its value in the absence of pulsations 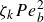 (3.5). The mean enhancement in diffusivity, normalised by its value in the absence of pulsations, is plotted figure 6(*a*), over a small range of wave-to-bulk Péclet number ratio, 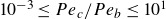 . The white dotted line in figure 6(*a*) indicates where shuttle dispersion is absent following (3.6). The critical Péclet number ratio 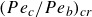 is shown by the black dotted line and follows (3.7), separating negative enhancement (blue) from positive enhancement (red) due to the pulsations. Along this black dotted line, the wave-induced dispersion enhancement vanishes (green region). Below the black dotted line, entropic slowdown dominates shuttle dispersion, indicated by the deep blue area. Entropic slowdown gets enhanced for smaller values of 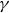 or for slender or tighter PVS geometries. Tighter PVS geometries and slower wave speeds relative to the bulk flow increase the residence time of the solute in the geometric constrictions, thereby enhancing entropic slowdown. Overall, entropic slowdown is most prominent for area ratios 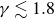 and for 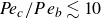 .

Figure 6(*b*) shows the diffusivity enhancement over a broader range of 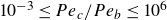 , relevant for the brain waves studied. The black dashed lines indicate the approximate upper and lower bounds of the physiological wave-to-bulk speed ratios. The abscissa is plotted on a logarithmic scale, and the colour contours of the diffusivity enhancement also scale logarithmically. For 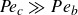 , from (3.5), the diffusivity enhances as 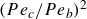 . The solid grey line in the plot indicates the limit 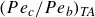 beyond which the long-time effective dispersion coefficients can be considered pre-asymptotic, with respect to transverse homogenisation following (3.8). The limit is representative and calculated using a PVS width of 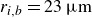 , 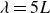 , 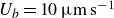 and 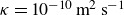 , yielding a value of 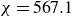 . The enhancement scales as 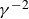 as evident from (3.5), and shown by the variation across the area ratios, except in regions where entropic slowdown dominates at low wave speeds and small area ratios in figure 6(*b*). This indicates that narrower annular geometries yield stronger enhancement. The black dashed vertical line indicate a representative delta wave, whereas the solid horizontal line indicates the area ratio 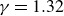 corresponding to a representative pial PVS used in most of the study. The white star indicates 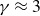 and 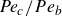 studied in § 3.3.4.

An expression for the average enhancement in solute drift can be similarly obtained by spatiotemporal averaging (3.4), and using Taylor expansions, as elaborated in Appendix E.2. The average solute drift can then be written as
(3.9)

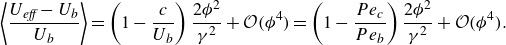




The prefactor 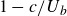 in the expression indicates the competition between the bulk flow and the wave speed in enhancing the solute drift. Solute drift is not enhanced when 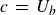 , implying the solutes simply ride along the bulk flow downstream when the wave speed matches the bulk flow. Any value of the wave speed 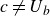 always results in an enhancement in solute drift. When 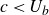 the enhancement in drift is positive downstream. The pulsations act as a pump reinforcing the bulk flow. When 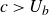 , the drift is enhanced towards the opposite direction of the fluid flow, implying that fast waves push the solute against the bulk flow and reverses the transport direction.

For the brain waves that we have studied 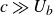 and the average drift is in the reverse direction of the bulk flow and linearly varies with 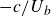 , that is faster waves lead to stronger drift in the upstream direction. This implies that, for a constant wavelength, the average drift scales linearly with frequency, and for a constant frequency, the average drift will scale linearly with wavelength. The linear dependence of drift on 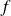 and 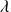 agrees with the nature of the contours of the mean drift enhancement plotted against the frequency and wavelength in figure 5(*b*). Similar to the diffusivity, the average drift also scales quadratically with the wave amplitude. This implies that small amplitude waves make negligible contribution to enhancing solute drift, but any change in amplitude can induce a second-order change in drift. The average drift is also related to the PVS area ratio with an inverse quadratic relation, implying that enhancement is suppressed in wide annuli with large 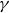 .

**Figure 7.**
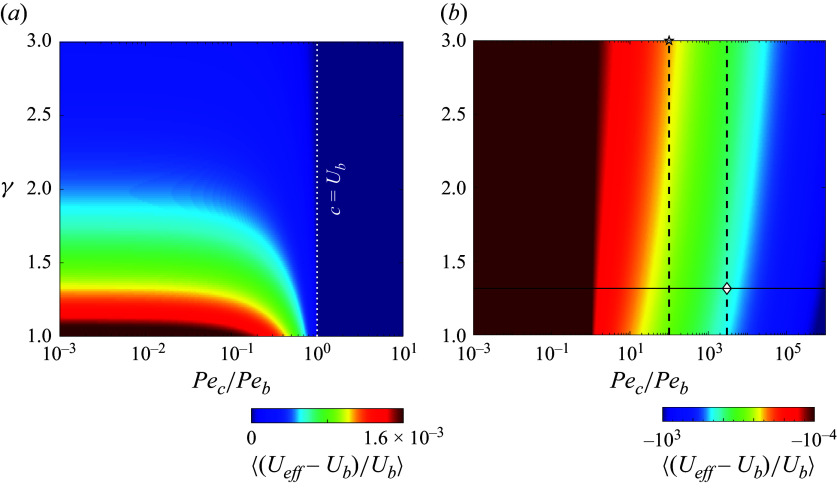
The contours of the mean enhancement in effective drift following (3.9), is shown for two different ranges of wave-to-bulk Péclet number ratio. (*a*) For 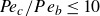 . The white dotted line indicates the speed ratio where solute drift is not enhanced. (*b*) For 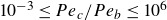 , with colours scaling logarithmically in the plot. The black dashed lines denote a representative physiological range of wave speeds 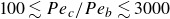 . The black solid horizontal line represents the area ratio 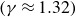 corresponding to the pial PVS simulations in figures 5 and 8, and the white diamond marker represents the waves with constant speed of 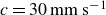 shown in figure 8. The white star indicates the area ratio 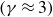 and Péclet number ratio 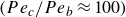 corresponding to penetrating PVSs as in table 2.

Figure 7 shows the mean enhancements in drift as functions of the area ratio and the wave-to-bulk Péclet number ratio, over both narrower and broader ranges of the latter, following the same conventions as figure 6. The mean enhancement is positive for 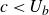 . The drift enhancement vanishes when 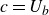 , as indicated by the white dotted lines in figure 7(*a*). For 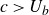 , the drift becomes negative, and for 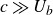 , it scales linearly with the wave-to-bulk speed ratio, consistent with (3.9).

The above results highlight the role of the wave parameters in modulating dispersion coefficient within the PVS. It is important to note, however, that we have used a constant amplitude 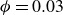 for all the cases, whereas physiological data suggest a dependency of the amplitude on the frequency. Nevertheless, the results provide fundamental insights into the enhancement of dispersion coefficients identifying regimes where the wave does not induce any enhancement, where it induces negative enhancement and where the wave-induced enhancement is quadratic positive. These regimes can be crucial in applications like externally applied neural stimulation (Cheng *et al.* 2020; Murdock *et al.* 2024), which may generate constant amplitude pulsations.

It is interesting to compare the enhancements in dispersion coefficients of representative low- and high-frequency cases that correspond to slow waves from figure 5. We find that for 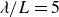 , low-frequency delta waves (shown by triangular symbol) yield lesser enhancement in diffusivity than high-frequency gamma waves (square symbol). Solute drift on the other hand is enhanced more in the upstream direction due to gamma waves. This is important because while delta waves are associated with CSF flow during non-rapid eye movement (NREM) sleep, gamma waves are typically associated with wakefulness heightened cognitive functions, like mediation and exercise (Hofle *et al.* 1997; Ding *et al.* 2016; Fultz *et al.* 2019; Bojarskaite *et al.* 2023). This implies that gamma waves may promote enhanced net transport in the PVSs. The enhancements induced by the gamma waves are related to the greater wave speed of gamma waves at a constant amplitude, enhancing 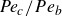 . Physiologically, however, this enhancement may be moderated if the amplitude diminishes with frequency or if the wave speed is constant, as discussed in the next section.

#### 3.3.3. Physiological enhancement of dispersion coefficients

We hypothesise that the amplitude of the radial deformation induced by the travelling waves is variable, and depends on their frequency. The hypothesis is physiologically grounded, since experiments on travelling waves have indicated that the power of a wave scales as the inverse of the frequency (Bojarskaite *et al.* 2023). Indeed, low-frequency delta waves have the larger amplitudes (Tononi & Cirelli 2006; Bojarskaite *et al.* 2023; Magazù, & Caccamo 2024). Since amplitude typically scales with the square root of power (Zhou *et al.* 2019; Mason, Barry & Clarke 2022; Ng, Jing & Westover 2022), we propose an empirical relation between the amplitude and the frequency of a brain wave
(3.10)

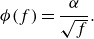

The coefficient 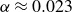 can be obtained by noting that 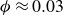 when 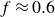 Hz in the experiments by Bojarskaite *et al.* (2023).

**Figure 8.**
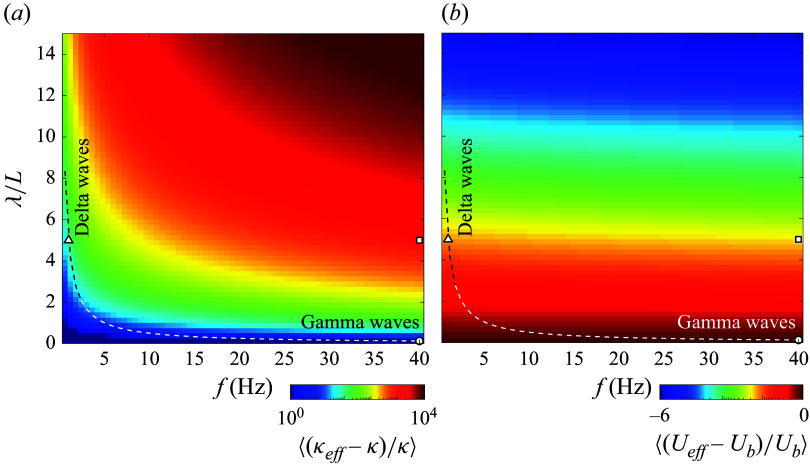
Mean enhancements of the Taylor–Aris dispersion coefficients for a solute in a slender pial perivascular segment with pulsations where the amplitude diminishes with frequency according to (3.10). All other parameters are identical to figure 5. Colour contours of (*a*) diffusivity enhancement and (*b*) drift enhancement, across the specified frequency and wavelength ranges. The dashed line represents a constant wave speed of 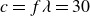 mm s^−1^. The white triangular, square and circular symbols are different cases simulated in figure 9. The colours scale logarithmically in (*a*).

Using the above relation, we plot the dispersion coefficient enhancements in figure 8. We note that the mean enhancement of diffusivity plotted in figure 8(*a*) is yet again maximised at higher frequencies and wavelengths, however, the absolute value is smaller in comparison with figure 5. As discussed earlier, for the range of frequencies of brain travelling waves, the mean enhancement in diffusivity scales as 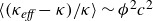 because of shuttle dispersion with the wave speed 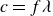 . When the amplitude varies with frequency, the scaling reduces to 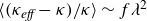 , implying linear dependence with frequency and quadratic dependence with the wavelength. The maximum value of 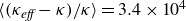 occurs at the largest frequency and wavelength studied.

The colour contours of the mean enhancement in drift are shown in figure 8(*b*). At large wave speeds, the mean enhancement scales as 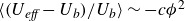 . Incorporating the amplitude–frequency relationship, the scaling reduces to 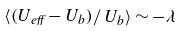 , implying a linear dependence with the wavelength and consequently an inverse dependence with frequency. The effect is evident in figure 8(*b*), where the magnitude of the mean drift enhancement increases with the wavelength. The maximum value of the enhancement is 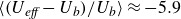 .

In mice, where most of the experimental progress in the glymphatic system has been made, the wavelength-to-domain length ratio is expected to lie in the lower bound of our study. Recent experiments demonstrate that cortical slow wave in mice organise themselves with a constant wave velocity of 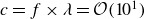 mm s^−1^ on average, under certain regimes like in wakefulness, under anasthesia or during sleep (Liang *et al.* 2021, 2023). This observation motivates our next hypothesis regarding physiological brain-wave organisation: Do the slow waves organise such that their wave speed is constant under a certain brain state? Since 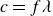 , this hypothesis implies higher-frequency waves produce a lower wavelength, and *vice versa*.

The dashed lines through the colour maps in figure 8 denote a constant wave speed of 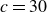 mm s^−1^, which lies towards the upper bounds of physiologically observed wave speed ranges in Liang *et al.* (2023). The wavelength corresponding to this wave speed, for a representative delta wave ( 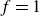 Hz), is 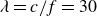 mm, which is equivalent to 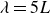 for the pial segment considered. Considering a constant wave speed combined with the amplitude–frequency relation, it is straightforward to show that the mean diffusivity enhancement scales as 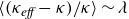 or equivalently, 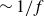 . This linear dependence on the wavelength and inverse dependence on frequency can be probed from the magnitude of the mean enhancement along the constant-speed line in figure 8(*a*). This implies that, at higher wavelengths, low-frequency delta waves enhance the diffusion coefficient by a factor of 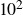 , whereas waves with frequency 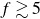 Hz barely contribute to the enhancement. The mean enhancement in drift has a similar behaviour to diffusivity, where the enhancement scales linearly with wavelength and inversely with frequency. This is evident by observing the constant-speed line in figure 8(*b*), indicating enhanced upstream drift at lower frequencies. We find that the drift enhancement is negligible for 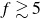 Hz.

**Figure 9.**
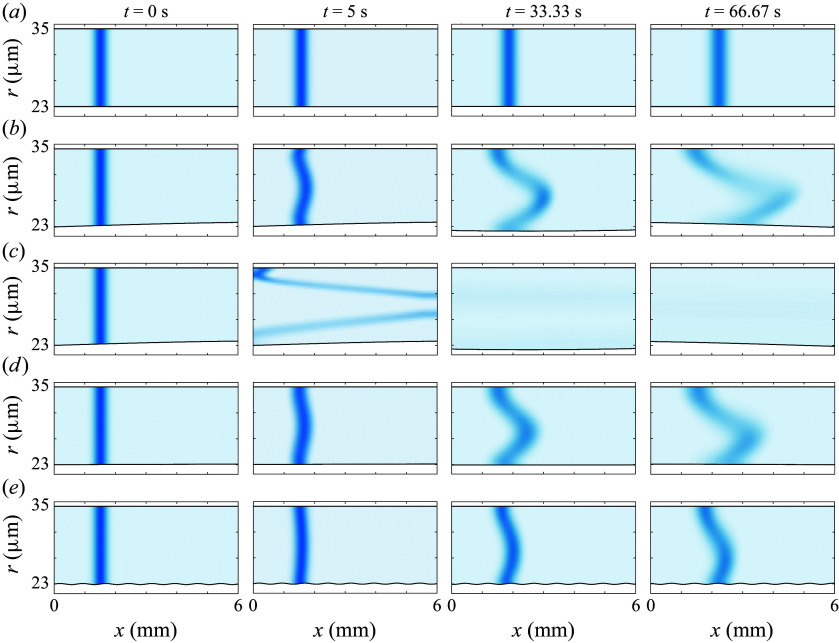
Solute concentration evolution in an annular domain subjected to radial deformations. The colour contours of 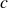 for (*a*) no radial deformation and radial deformation corresponding to brain waves in the rest of the panels. Radial deformations corresponding to (*b*) a delta wave with 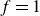 Hz, 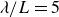 and 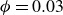 , (*c*) a gamma wave with 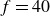 Hz, 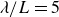 and 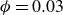 , (*d*) a gamma wave with 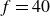 Hz, 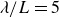 and 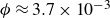 and (*e*) a gamma wave with 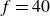 Hz, 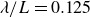 and 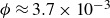 . The radial axis is exaggerated for the ease of visualisation ( 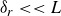 ). Relevant simulation parameters: 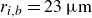 , 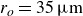 , 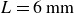 , 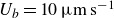 , 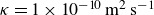 . The colour varies in the range 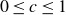 . The wave period is 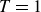 s in panel (*b*) and 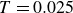 s in panels (*c*–*e*).

In order to visualise the solute-evolution dynamics resulting from the above-mentioned physiological mechanisms of radial deformation, we plot the colour contours of the solute concentration field in figure 9. The solute concentration fields are obtained by solving the advection–diffusion (2.2) using a finite-difference approach that is implemented in Matlab. The details of the numerical approach are provided in the Appendix C.

Figure 9(*a*) plots the colour contours of the solute concentration field at four different times, for the case of no pulsations. The panels show the temporal evolution of the concentration field with time increasing from left to right. The concentration field is initiated as a Gaussian at 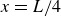 of the form provided in Appendix C. The boundary conditions are no flux on all the material walls. As time progresses, axial spreading of the concentration field results from the shear-induced deformation by the parabolic flow profile in the background, which induces cross-sectional homogenisation of the concentration field.

Figure 9(*b*) plots the concentration fields in the domain with pulsations corresponding to a representative delta wave with 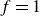 Hz and 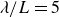 , with an amplitude of 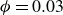 . The dispersion enhancements in this scenario correspond to the white triangular symbol in the colour maps of dispersion coefficients plotted in figure 5. The mean diffusivity enhancement is expected to be 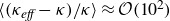 . This is evident from the concentration fields, where the shear-induced deformation induced by the pulsatile flow yields rapid axial spreading of the concentration field via shuttle dispersion. The drift enhancement is expected to be 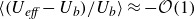 , or a weak upstream drift on an average of the concentration field.

It is important to note that, since the drift represents the time rate of change of the centroid of the concentration (as defined in (C2) and (C5)), the travelling wave induces, on average, a negative displacement of this centroid. However, the axial flow profile remains parabolic on average. Near its centre, the concentration field is stretched out in the downstream direction by the positive bulk flow, with the effect of the wave on the drift only significant to 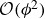 . Near the top and bottom walls, the weak negative drift induces axial stretching in the upstream direction. The enhanced diffusivity also leads to greater axial spread. The axial stretching of the concentration field is evident from comparing figure 9(*b*) with the case of no pulsations in figure 9(*a*). Additionally, the solute concentration at the bottom wall lingers longer compared with the top wall, because of the transient trapping of the solutes by the radial velocity induced at the bottom wall.

Figure 9(*c*) plots the identical scenario presented in figure 9(*b*) but for a representative gamma wave with 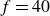 Hz, 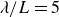 . The amplitude is kept constant at 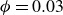 , similar to the delta wave. The regime corresponds to the white square symbols in the colour maps of the dispersion coefficient enhancement in figure 5. The strong upstream drift and the large enhancement in diffusivity in this scenario induces rapid axial spreading of the concentration field. The centre of the concentration field is stretched out in the downstream and transported out of the domain. The concentration field near the top and bottom walls is transported upstream and exits from the left boundary.

Figure 9(*d*) plots the frequency-dependent amplitude scenario for parameters corresponding to a gamma wave in figure 9(*c*). As the frequency increases within the gamma range, the amplitude decreases to 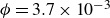 according to (3.10). The corresponding regime in dispersion coefficients is shown in figure 8 by the white square symbol. The weak amplitude leads to moderate axial stretching of the concentration profile, and induces a weak upstream drift, in agreement with the values predicted in figure 8. Finally, the concentration fields of the solute for the constant-wave-speed scenario is shown in figure 9(*e*). Here, maintaining a constant wave speed reduces the wavelength to 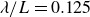 for a representative gamma wave with 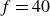 Hz. The corresponding dispersion enhancements are shown by the circular data points in figure 8, with the dashed line plotting 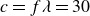 mm s^−1^. The dispersion enhancement is negligible in this scenario, as evident from the moderate axial stretching and drift of the concentration profile, and nearly identical to figure 9(*a*).

**Figure 10.**
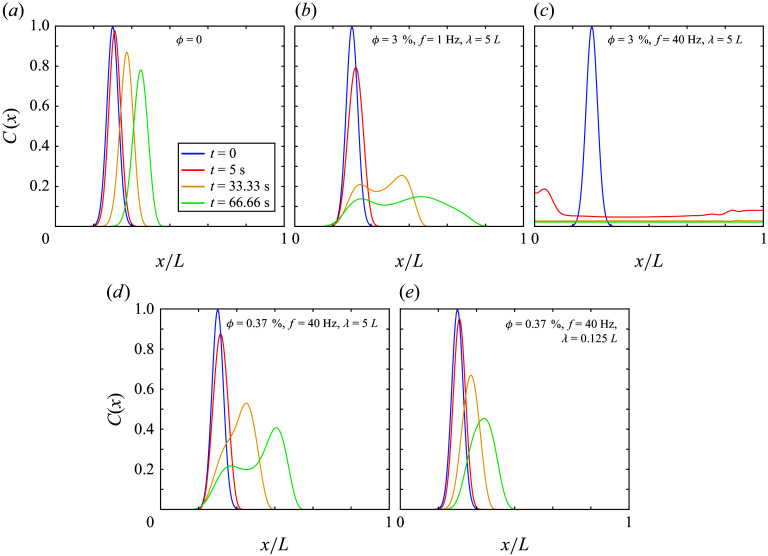
The cross-sectionally averaged solute concentration profile 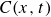 as a function of the axial extent 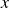 corresponding to the contours of the concentration shown in figure 9. The different times are indicated by the colours labelled in panel (*a*). We show 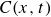 as a function of 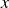 in a domain with: (*a*) no radial deformation 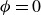 , (*b*) deformations corresponding to a representative delta wave, (*c*) a gamma wave, (*d*) a gamma wave with frequency-dependent amplitude and (*e*) a gamma wave with frequency-dependent amplitude and constant wave velocity of a delta wave at 30 mm s^−1^. The radial diffusion timescale 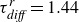 s for all cases. The radial diffusion timescale 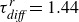 s for all cases. The wave period is 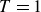 s in panel (*b*) and 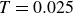 s in panels (*c*–*e*).

We next plot the cross-sectionally averaged concentration 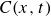 as a function of 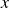 in figure 10. When pulsations are absent, the Gaussian profile used for initiation at 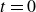 , is preserved with time, as expected in classical Taylor–Aris dispersion. This is shown in figure 10(*a*), where the radial diffusion timescale is 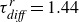 s. For pulsations with 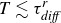 , since the cross-sectional homogenisation does not get completed within a pulsation cycle, we observe deviations from the Gaussian profiles in figure 10(*b*–*d*). The departure from the Gaussian profile is moderate in figure 10(*b*) with 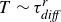 and becomes more pronounced in figure 10(*c*), where 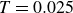 s, corresponding to the regime that is pre-asymptotic with respect to cross-sectional homogenisation of the solute. The departure from the Gaussian profile is reduced in figure 10(*d*) because of the frequency-dependent amplitude, even with 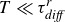 . Interestingly, we find the profiles to be approximately Gaussian again in figure 10(*e*) even if the regime is pre-asymptotic. This is because the dispersion coefficient enhancement is moderated by the frequency-dependent amplitude, as well as the constant imposed wave speed. Together, these effects produce small wavelengths, weak amplitude and consequently negligible enhancement in dispersion coefficients.

#### 3.3.4. Analysing dispersion enhancement across murine brain states in an experimentally measured penetrating PVS

We now extend our analysis of dispersion coefficient enhancement on experimental data of pulsations of a penetrating PVS of mice from Bojarskaite *et al.* (2023). The penetrating PVS and vasculature data in the experiment were measured using two-photon microscopy on naturally sleeping mice. We extract the base lumen radii, wave amplitudes and PVS widths across eight different brain states corresponding to: wake before sleep (WBS), NREM, intermediate sleep (IS), rapid eye movement (REM), wake after sleep (WAS), quiet wakefulness (QW), whisking (whisk) and locomotion (loco). We note that both 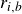 and 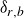 change across these brain states in the experiment. Additionally, there are significant variations in the wave amplitude 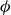 across these states.

We specifically focus on the effect of low-frequency waves ( 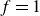 Hz, and 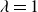 mm) on the dispersion coefficients in these regimes. We model a travelling radial pulsation wave (2.1) with a wave speed of 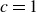 mm s^−1^, which lies towards the lower ends of physiologically observed wave-speed ranges (Liang *et al.* 2023). With penetrating PVS lengths varying from 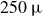 m to 1 mm (Asgari, de Zélicourt & Kurtcuoglu 2016; Kedarasetti *et al.* 2022; Bojarskaite *et al.* 2023), this yields a wavelength-to-domain length ratio varying in the range 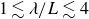 . We hold the bulk-flow speed constant at 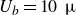 m s^−1^, yielding a Péclet number ratio of 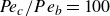 . Our goal here is not to reproduce the exact waveform measured experimentally, but to demonstrate that our theoretical framework provides a flexible approach that can be readily applied to physiologically measured pulsation data.

The effective dispersion coefficients for this wave can be obtained from ((3.3)–(3.4)) from our analysis. The resulting enhancements in the dispersion coefficients are summarised in table 2. We find that the enhancement in dispersion coefficients is modest, with a maximum of approximately 5 % for drift and 1 % for diffusivity, over the molecular diffusivity. When normalised with the bulk-flow dispersion, the diffusivity enhancement, induced only by the wave, is 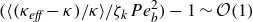 , indicating that the wave induces a correction comparable to steady bulk-flow-induced dispersion.

**Table 2.** Enhancements in effective dispersion coefficients for a solute in a penetrating PVS for a representative delta waves ( 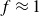 Hz, 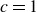 mm s^−1^ and 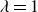 mm) and sinusoidal wall deformation (2.1) but with experimental values of lumen radii, PVS width and wave amplitude, during various brain states, namely WBS, NREM, IS, REM, WAS, QW, whisk and loco in mice, determined from supplementary tables 3, 4, 7 and 8 from Bojarskaite *et al.* (2023). The solute considered is amyloid beta with 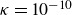 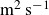 , the bulk-flow velocity is 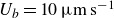 , 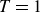 s and 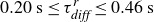 .

	WBS	NREM	IS	REM	WAS	QW	Whisk	Loco
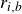 ( 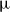 m)	5.95	6.1	6.6	7.75	5.65	5.7	6.15	6.65
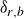 ( 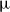 m)	6.2	6.1	5.6	4.5	6.8	6.5	5	5.5
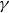	3.17	3	2.42	1.5	3.86	3.58	2.9	2.34
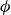 (%)	2.5	4.1	3.4	1.3	1.8	3.2	2.5	3.5
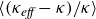 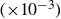	4.33	8.87	7.74	2.48	3.34	5.55	4.47	8.25
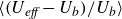 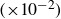	−1.22	−3.68	−3.90	−1.49	−0.427	−1.57	−1.46	−4.42

The comparatively small enhancement, relative to the pial PVS regimes considered earlier (for instance figure 8), arises due to several contributing factors. Firstly, the wave speed studied is 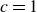 mm s^−1^ compared with 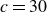 mm s^−1^ in pial PVS, resulting in reduced enhancements that scale as 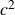 . Secondly, the penetrating PVS have wider geometries, with 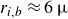 m and 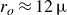 m, yielding a mean area ratio of 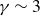 . Since enhancements scale as 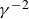 , larger 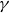 yields weaker wave-induced enhancements. Finally, the smaller PVS gap for the penetrating PVS (approximately 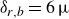 m compared with 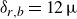 m for the representative pial PVS) reduces the bulk-flow-induced diffusivity 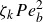 . Table 2 shows that, across the different brain states, the variation in enhancements is driven by change in geometry and pulsation amplitudes, consistent with the scaling 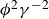 for fixed 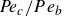 ((3.5) and (3.9)). The white stars in figures 6(*b*) and 7(*b*) represent the penetrating PVS results, characterised by larger 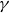 and smaller 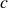 , while the diamond symbols represents the pial PVS results. Although the diffusivity enhancement is largest during the NREM sleep state, in qualitative agreement with Bojarskaite *et al.* (2023), the magnitude of enhancement is sensitive to the chosen wave speed. For instance, if the wave speed was increased from 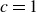 to 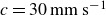 , which is the upper bound of the physiological travelling waves in mice, for the NREM case, the diffusivity enhancement induced by the wave relative to bulk flow would increase to 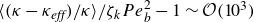 . These estimates highlight how physiological parameters can be incorporated in our theoretical framework.

### 3.4. Discussion

Overall, we find that the enhancement in dispersion coefficients is governed by the wave-to-bulk Péclet number ratio 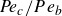 and the PVS area ratio 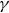 . In the limit of 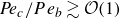 , the effective diffusivity and drift enhancement induced by the wall deformation wave scale as
(3.11)

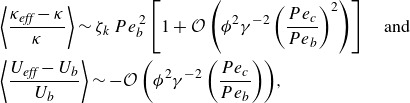

implying stronger enhancements, induced by the wave, for narrower PVSs and for waves faster than the bulk flow (figures 6–7). On the other hand, for 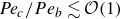 , we find entropic slowdown in diffusivity and positive downstream drift.

For a representative pial PVS with 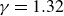 and a delta wave ( 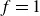 Hz, 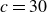 mm s^−1^, 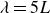 ), yielding 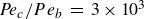 , we get a diffusivity enhancement 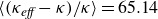 over the molecular diffusivity, and a drift enhancement yielded of 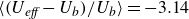 . When normalised by the bulk-flow contribution, the wave-induced enhancement of diffusivity is 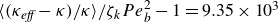 . At higher frequencies, physiological mechanisms like frequency-dependent amplitude (3.10), and approximately constant wave velocity reduce the enhancement (figure 8). For instance, a representative gamma wave in the pial PVS geometry yields a wave-induced diffusivity enhancement of 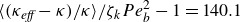 while drift enhancement of 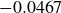 .

In contrast, for a representative penetrating PVS with 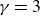 subjected to delta wave pulsations with a low wave speed of 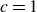 mm s^−1^ (yielding 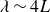 ) and 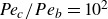 (§ 3.3.4), we find a maximum enhancement of 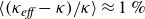 for effective diffusivity over the molecular value, and 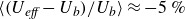 for effective drift. The reduction arises because of lower wave speeds, smaller PVS gaps 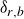 , but with larger values of 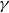 (star symbols in figure 6–7).

It is useful to compare our results with previous studies on dispersion enhancement in PVSs. Troyetsky *et al.* (2021) used an oscillating axial pressure gradient and found a diffusivity enhancement of 5 % over the molecular diffusivity in an annular geometry with 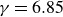 and 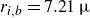 m subjected to oscillation of 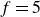 Hz, representative of cardiac pulsations. With identical parameters, our model yields comparable magnitudes, for wave speed of 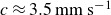 (corresponding to 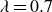 mm) and amplitude of 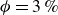 . Sharp, Carare & Martin (2019) reported diffusion enhancement of 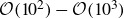 over free diffusion for 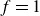 Hz, for non-porous periarterial channels, with porous channels inducing lesser enhancement. These magnitudes are consistent with our model predictions for 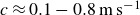 . It is useful to note that our flow generation mechanism differs from the above studies of pressure-driven dispersion, as our pressure field is not externally imposed, but emerges as a consequence of the spatiotemporal variations of the inner radius 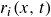 .

Troyetsky *et al.* (2021) also evaluated the enhancements from Asgari *et al.* (2016) to be 27 %–110 % over molecular diffusivity in a porous PVS model ( 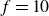 Hz, 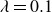 m, 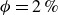 , 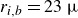 m and 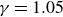 ). For identical parameters, our open-annulus model calculates a diffusion enhancement of 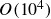 over the molecular diffusivity. This difference may be attributed to the fact that Asgari *et al.* (2016) uses a porous medium with a porosity of 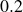 while we treat the PVSs as open annular conduits. Finally, the fluid–structure interaction simulations by Bojarskaite *et al.* (2023) employed the exact waveform from their experiments, and found diffusivity enhancement by 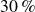 for 70 kDa tracers ( 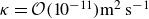 ), and 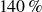 for 2000 kDa tracers ( 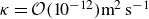 ), due to low frequencies of 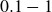 Hz during the NREM sleep state. Our model determines an enhancement of 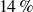 and 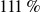 over the free diffusion for the 70 and 2000 kDa tracers, respectively, for an identical PVS geometry using an ideal sinusoidal radial deformation with 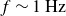 , 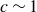 mm s^−1^.

## 4. Conclusions

We have used asymptotic expansions to quantify the influence of travelling radial deformations on the effective dispersion coefficients of a solute in an annular segment that is subjected to spatiotemporal deformations in its inner boundary. The domain replicates a segment of PVS in the brain subjected to wall deformations that correspond to travelling waves in the brain. We have presented a careful study that builds in complexity from analysing the dispersion coefficients in a domain without pulsations to quantifying the dispersion behaviour in different physiological regimes spanned by the wavelength and frequency of the deformation. Our intention was to quantify parameter regimes that enhance or suppress dispersion in slender pulsating annular channels. Our approach has allowed us to probe the complex dependence of the dispersion coefficients on the wave parameters in PVSs, where many fundamental theoretical questions remain. We analyse these questions at a level of quantitative detail that is currently not possible experimentally.

We have shown that perivascular solute dispersion is dominated by shuttle dispersion induced by travelling waves that deform the arterial lumen at speeds much faster than the bulk-flow speed. The mean enhancements in diffusivity and drift scale as the square of the wave amplitude. We also observe entropic slowdown of diffusivity at wave speeds comparable to the bulk-flow speed. We explore physiologically relevant regimes, such as frequency-dependent amplitude and constant wave speed of brain pulsations, which can moderate dispersion at higher frequencies. The travelling wave considered in this study is a sinusoidal wall deformation. However, the analytical approach developed can be extended to accommodate arbitrary forms of the travelling pulsatile wave.

A physical understanding of these fundamental contributions to the dispersion dynamics will be useful on several levels. The identification of the important features will guide the development of the theoretical ideas necessary to describe the dispersion dynamics of solutes in eccentric annular PVSs, branching PVSs, porous PVSs and incorporating a compliant and absorbent outer wall. For instance, a recent study showed that selective permeability of the channel walls to fluid, but not solute, can reduce effective dispersion (Gan *et al.* 2025). Incorporating an identical boundary condition in our theoretical framework would be an interesting direction in the future. Our results overall suggest that it may be possible to control the dispersion behaviour by modulating the properties of the travelling deformation wave. Control of dispersion in this sense could be achieved by the use of externally applied neural stimulation that drive pulsations of particular wave speeds across a PVS.

**Table 3.** List of parameters used in this paper.

Dimensional parameters	Non-dimensional parameters
Inner radius, 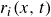	Normalised inner radius, 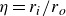
Outer radius, 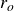	Normalised base inner radius, 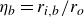
Base inner radius, 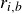	Area ratio, 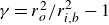
Annular thickness, 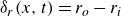	Local Péclet number, 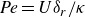
Base annular thickness, 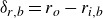	Bulk-flow-induced Péclet number, 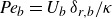
Domain length, 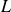	Relative amplitude, 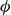
Wavelength of travelling wave, 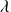	PVS aspect ratio, 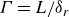
Frequency of travelling wave, 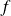	Phase of wave, 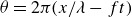
Wave velocity, 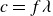	Wave Péclet number, 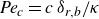
Cross-sectionally averaged axial velocity, 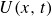	Solute concentration, 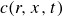
Bulk velocity, 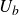	Cross-sectionally averaged solute concentration, 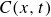
Centroid of the solute, 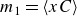	Mean solute concentration, 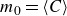
Variance of the concentration profile, 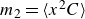	Wavelength transport parameter, 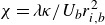
Molecular diffusivity, 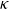	Wavenumber, 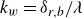
Effective diffusivity, 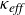	Wave-induced *Re*, 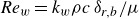
Solute drift, 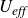	Womersley number, 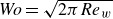
Radial diffusion timescale, 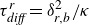	Stokes number, 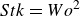
Axial advection timescale, 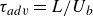	Perturbation expanded terms of 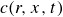 , 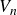
Wave induced timescale, 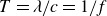	Calibration coefficient for physiological relation between 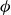 and 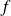 , 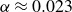 (Bojarskaite *et al.* 2023)
Small parameter for perturbation expansion, 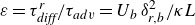	—
Flow rate, 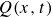	—
Steady part of the flow rate, 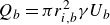	—
Annulus cross-sectional area, 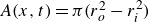	—
Perturbation expanded terms of 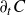 , 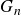	—
Log-polynomial function in 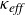 expression, 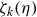	—
Log-polynomial functions in 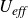 expression, 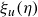 , 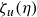 , 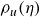	—

## Figures and Tables

**Figure 11.**
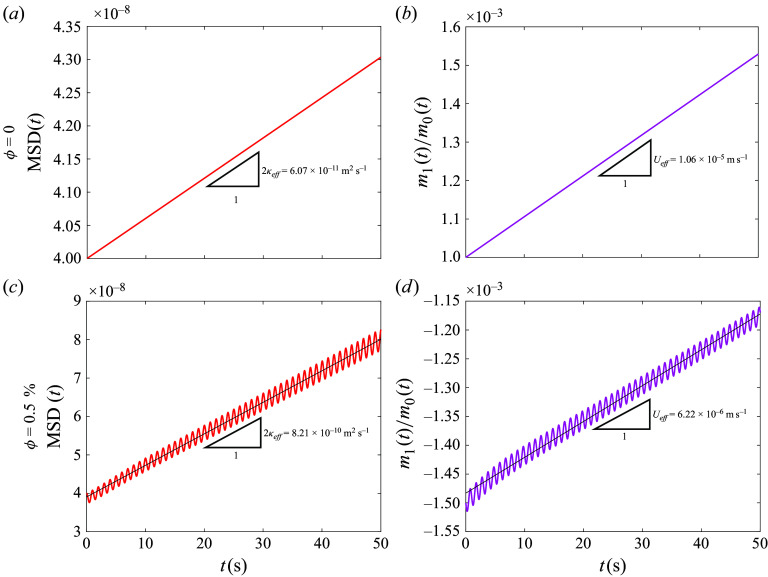
Comparison of the effective diffusivity and drift obtained from finite-difference simulations and analytical equations based on Aris’s solution (Appendix F). (*a*) Temporal evolution of the MSD obtained from the finite-difference simulations. (*b*) Temporal variations of the centroid of the solute concentration 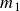 normalised with the mean solute concentration 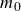 . (*c*) The MSD as a function of time for a pulsating wall 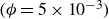 case for amyloid beta monomers 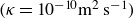 , obtained from the finite-difference simulations. (*d*) Value of 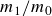 as a function of time for the same case as (*c*). Relevant parameters: 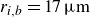 , 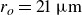 , 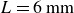 , 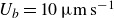 . Wave frequency for panels (*c*) and (*d*) is 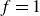 Hz.

## Appendix A. List of parameters used in the study

We present a comprehensive summary of the different symbols and parameters used in this paper in table 3.

## Appendix B. Derivation of the flow profile in a slender annulus subjected to weak spatiotemporal fluctuations

We begin with the governing equations of momentum for an incompressible flow in an annular domain in the axisymmetric formulation

(B1)

(B2)

We next non-dimensionalise these equations with the appropriate scales, which include the wave parameters governing the wall pulsations, such as wavelength  , wave speed  and wave period,

(B3)

The non-dimensionalised axial momentum equation yields

(B4)

(B5)

(B6)

(B7)

while the non-dimensionalised radial momentum yields

(B8)

(B9)

(B10)

(B11)

We obtain two non-dimensional parameters

(B12)

(B13)

The wavenumber  , defined here, physically interprets the inner wall curvature of the annular conduit. The wave-induced Reynolds number  is the ratio of the timescale of viscous diffusion in the transverse direction  to the wave passage time over one wavelength, or simply, the wave period  . It is also straightforward to obtain a relation between  and the Womersley number defined using the radial length scale,  , and the Stokes number,  , such that

(B14)

The typical width of PVS is considerably smaller than the wavelength of the wall pulsations  . For the parameters used in this study, we find the maximum value of the wavenumber to be  . Additionally, the deformation amplitude is weak  , ensuring both the instantaneous and baseline values of  remain smaller than the wavelength. With  , the radial momentum (B11) simplifies to

(B15)

Thus the pressure is independent of the radial direction. Indeed, this is the well-known lubrication theory approximation which has been widely used in the context of pulsatile wall-induced flow in slender conduits (Shapiro, Jaffrin & Weinberg 1969; Li & Brasseur 1993). Additionally, in the ranges of physiologically informed wave speeds  and wavelengths  considered for the current study, we get a maximum wave-induced Reynolds number of  , which enables us to assume that inertial effects are negligible;  is in agreement with past studies which have shown that the Womersley number of CSF flow through the spinal canal and the PVS is much less than 1 (Sánchez *et al.* 2018; Kelley & Thomas 2023). These limits (  ,  ) are indeed reasonably good global approximations even when  and  are finitely small (Jaffrin 1973; Takabatake & Ayukawa 1982; Takabatake, Ayukawa & Mori 1988). With the above limits, the left-hand side of (B7) and the term  on the right-hand side can thus be omitted, to yield the following reduced equation:

(B16)

We can now dimensionalise (B16), and apply standard treatment of no-slip boundary conditions at the inner and outer radii (White 2006), yielding the quasi-Poiseuille velocity profile

(B17)

that is driven by a spatiotemporally varying axial pressure gradient  induced by the wall pulsations.

## Appendix C. Validation of effective dispersion coefficients using solute transport simulations in a segment of PVS

In the manuscript, we have presented a validation of the effective dispersion coefficient for a case with no pulsations,  , in figure 3, where we found reasonable agreement with the analytical solution by Aris (1959) (elaborated in Appendix F). In order to validate the dispersion coefficients in the case of PVS with pulsations, we conduct finite-difference simulations of the advection–diffusion equation governing the solute dynamics (2.2). Our finite-difference numerical simulations are in two-dimensional and yield  , however, to compare with theory we compute the cross-sectionally averaged concentration  from the simulated concentration fields. The mean solute concentration,  , centroid of the solute,  and the variance of the solute concentration profile,  , are given as

(C1)

(C2)

(C3)

where  with  is the pth moment in Aris’s method of moments (Aris 1956, 1959).

Multiplying  to the both sides of the one-dimensional advection–diffusion equation governing  (2.3) and integrating it along the length, we obtain

(C4)

Using the boundary condition that the solute concentration never reach the ends of the domain  , the equation can be reduced to

(C5)

This relationship indicates that the effective advection coefficient of a solute can be obtained by computing the slope of the line that fits  versus

(C6)

Similarly, we can obtain the expression for  by multiplying  with both sides of (2.3), integrating along the length and applying the boundary conditions

(C7)

The MSD of a passive solute at time  , relative to a reference position  , is defined as a quantitative measure of the deviation of the position of a particle from the reference position over time

(C8)

Choosing the reference as the instantaneous mean position,  yields

(C9)

Performing the time derivative of the MSD, (C9), we obtain a relation with the effective diffusion coefficient

(C10)

Thus, the slope of the line that fits the temporal variation of MSD is equal to twice the effective diffusion coefficient.

We next solve for the effective dispersion coefficient using finite-difference simulations of (2.2) and computing the moments and MSD of the solute concentration in Matlab. Due to the flow reversals induced by wall-driven pulsations, characterised by oscillatory to-and-fro motion, a second-order upwind scheme is employed. A first-order explicit Euler Runge–Kutta scheme time integration scheme was implemented. The boundary condition is no flux on the material walls, and the initial condition is a Gaussian distribution of the solute, given by  , where  is the standard deviation,  and  is the domain length. The Gaussian initial condition is identical to the concentration profile shown in figure 9(*a*) at  . The numerical simulations are implemented in Matlab. Two different cases have been considered; one with no wall pulsations,  , and another with spatiotemporally pulsating inner walls with  . The solute considered has a diffusivity of  , which is comparable to amyloid beta.

Figures 11(*a*) and 11(*b*) show the case without pulsations. The base PVS width is  and the Péclet number, computed using the maximum fluid velocity, is 2. The temporal evolution of MSD is plotted in figure 11(*a*). The MSD increases linearly with time and the effective diffusion coefficient can be obtained by computing the slope of the line and dividing by two, yielding  , which is comparable to the effective diffusion coefficient from the solutions by Aris (Appendix F) (Aris 1959; Troyetsky *et al.* 2021), which predict a diffusivity of  . The temporal evolution of  is shown in figure 11(*b*). The slope of the line returns a value of  m s^−1^, which is comparable to the mean fluid velocity of the steady parabolic flow profile,  .

Figures 11(*c*) and 11(*d*) show the case of a PVS subjected to pulsations induced by a low-frequency travelling wave with  . We compare the computed diffusivity and drift obtained from the simulations with the long-time effective expressions. The pulsations induce oscillations to the computed MSD, which still increases linearly, on average, with time, as shown in figure 11(*c*). The best-fit line to the distribution is shown by the dashed line in black, the slope of which is expected to correspond to twice the effective diffusion coefficient  . For this case, we get an effective diffusion coefficient of  from the finite-difference simulations, which is approximately  more than  , the diffusion coefficient obtained from the asymptotic analysis.

Figure 11(*d*) shows the temporal evolution of  . The slope of the best-fit line to the data yields a value of   m s^−1^ which under-predicts the effective advection coefficient obtained from the asymptotic analysis by  . Several factors, like Péclet number, and the numerical approach implemented contribute to the deviations observed between the computed and the asymptotically obtained dispersion coefficients. The finite-difference approach induces several non-physical errors like numerical diffusion and truncation errors, implying that the order of accuracy of the numerical approach, as well as factors like grid spacing when computing the integral for the moments, contribute to the deviation. A key challenge is to resolve the fast radial diffusion. Additionally, the first moment may be strongly sensitive to the finite-domain boundary. Further errors can be attributed to the exponentially small terms arising from the initial displacement of the centroid of the Gaussian profile in the simulations, which is expected to deviate from the theory, that assumes a well-mixed tracer (Young & Jones 1991). For the case with pulsations, this error may amplify because of transient displacements of the solute centroid resulting from pulsations.

## Appendix D. Expressions of the functions obtained from the asymptotic expansion

The expanded expressions of the spatiotemporal functions  ,  ,  and  referenced in (3.1) are given as

(D1)

(D2)

(D3)

(D4)

Substituting the expressions for  and  in (3.1) yields  . Accordingly, the non-dimensional, cross-sectionally averaged solute concentration  in a straight annular channel, with a pulsating inner radius  , a fixed outer radius  and a parabolic axial velocity profile, characterised by a radially averaged flow velocity  , is approximated by

(D5)

and returning to dimensional form, we arrive at (3.2), given as

(D6)

Simplifying and reorganising the terms in this equation, we get

(D7)

(D8)

**Table 4.** Calculated coefficients of the effective advection and diffusion expression terms.

**p**				
**0**				
**1**				
**2**				
**3**				
**4**				
**5**				
**6**				
**7**			—	
**8**			—	
**9**			—	
**10**			—	
**11**			—	
**12**			—	
**13**	—	—	—	
**14**	—	—	—	
**15**	—	—	—	
**16**	—	—	—	

Redefining the functions as  ,  and  , where  , we can rewrite the expressions for  and  as in § 3.1, given as

(D9)

(D10)

In the above, we neglect the term associated with  . The coefficients of  are of the orders of  and lower, rendering its contribution negligible compared with the rest of the terms in the equation. The respective expressions for the redefined  ,  ,  and  are given as

(D11)

(D12)

(D13)

The functions  ,  and  are given by log-polynomial expansions of  , the ratio of instantaneous inner radius to the outer radius. The polynomial order is  . The coefficients obtained in these expressions,  to  , are reported in table 4.

## Appendix E. Derivation of mean dispersion coefficients

### E.1. Mean diffusivity enhancement

Consider the cross-sectionally averaged velocity equation, given in (2.12), as follows:

(E1)

This can be simplified to the following equation under the assumption  , given as:

(E2)

The expression of the dimensionless quantity,  used in (3.3), called the form factor, is defined in (D13). Multiplying  with  , where the annular thickness is defined as

(E3)

dividing with  and taking the phase average of the whole term, we get

(E4)

(E5)

where  and  . This expression excludes the means of odd powers of  since  when  is odd. The expression for  is expanded around  , given as

(E6)

(E7)

since  . The averages used in (E5) above are expressed as

(E8)

(E9)

Replacing these terms in (E5), we get the averaged diffusion enhancement as

(E10)

(E11)

This is the same expression of the phase-averaged diffusion enhancement given in (3.5).

### E.2. Mean solute drift enhancement

We lead from the equation of the effective advection (3.4) by dividing it with  and subtracting  as follows:

(E12)

We purposefully neglect the third term on the right-hand side of (3.4) since the coefficients of  are of the orders if  and lower. Taking the mean of this equation, we get

(E13)

and the phase averages required in the above equation are defined as

(E14)

(E15)

(E16)

(E17)

(E18)

The second and third terms are zero since  when either of  or  is odd. Thus, the advection enhancement takes the form

(E19)

(E20)

## Appendix F. Analytical solution of diffusivity enhancement in an annular domain

The enhancement term for the lateral diffusion  in an annular geometry with  and  as the outer and inner radii, respectively, given by Aris (1959) is

(F1)

where  is the mean flow velocity of the fluid, and  , where

(F2)

(F3)

(F4)

where  ,  and  . Hence, the effective diffusion is given as  without any component of oscillatory flow (Troyetsky *et al.* 2021).
